# CCL2-CCR2 axis recruits tumor associated macrophages to induce immune evasion through PD-1 signaling in esophageal carcinogenesis

**DOI:** 10.1186/s12943-020-01165-x

**Published:** 2020-02-27

**Authors:** Hui Yang, Qiannan Zhang, Miao Xu, Lei Wang, Xuewei Chen, Yongquan Feng, Yongning Li, Xin Zhang, Wenming Cui, Xudong Jia

**Affiliations:** 1 NHC Key Laboratory of Food Safety Risk Assessment, China National Center for Food Safety Risk Assessment, No.7 Panjiayuan Nanli, Beijing, 100021 China; 2 West China School of Public Health, Sichuan University, Chengdu, 610041 China; 3 Affiliated Hospital of Jining Medical University, Jining, 272001 China; 4 Department of Operational Medicine, Tianjin Institute of Environmental and Operational Medicine, Tianjin, 300050 China

**Keywords:** CCL2, CCR2, Carcinogenesis, Esophageal squamous cell carcinoma (ESCC), Programmed death-1(PD-1), Tumor associated macrophages (TAMs)

## Abstract

**Background:**

The poor prognosis of esophageal squamous cell carcinoma (ESCC) highlights the need for novel strategies against this disease. Our previous study suggested the involvement of CCL2 and tumor associated macrophages (TAMs) in esophageal carcinogenesis. Despite the recognition of TAMs as a promising target for cancer treatment, mechanisms underlying its infiltration, activation and tumor-promotive function in ESCC remain unknown.

**Methods:**

Human esophageal tissue array and TCGA database were used to evaluate the clinical relevance of CCL2 and TAMs in ESCC. F344 rats and C57BL/6 mice were treated with N-nitrosomethylbenzylamine (NMBA) to establish orthotopic models of esophageal carcinogenesis. CCL2/CCR2 gene knockout mice and macrophage-specific PPARG gene knockout mice were respectively used to investigate the role of infiltration and polarization of TAMs in ESCC. CCL2-mediated monocyte chemotaxis was estimated in malignantly transformed Het-1A cells. THP-1 cells were used to simulate TAMs polarization in vitro. RNA-sequencing was performed to uncover the mechanism.

**Results:**

Increasing expression of CCL2 correlated with TAMs accumulation in esophageal carcinogenesis, and they both predicts poor prognosis in ESCC cohort. Animal studies show blockade of CCL2-CCR2 axis strongly reduces tumor incidence by hindering TAMs recruitment and thereby potentiates the antitumor efficacy of CD8^+^ T cells in the tumor microenvironment. More importantly, M2 polarization increases PD-L2 expression in TAMs, resulting in immune evasion and tumor promotion through PD-1 signaling pathway.

**Conclusion:**

This study highlights the role of CCL2-CCR2 axis in esophageal carcinogenesis. Our findings provide new insight into the mechanism of immune evasion mediated by TAMs in ESCC, suggesting the potential of TAMs-targeted strategies for ESCC prevention and immunotherapy.

## Background

Esophageal cancer is the sixth most common cancer in the world. It was estimated that over 570,000 cases occur and nearly 510, 000 died in 2018 worldwide [1]. Most of cases are found in Eastern Asia and Europe, which respectively account for 76.3 and 10.2% of the five-year prevalence [1]. In histology, although the incidence of esophageal adenocarcinoma (EAC) is increasing in Western countries, esophageal squamous cell carcinoma (ESCC) is still the predominant type, accounting for 90% of all esophageal cancer cases [2, 3]. Due to the lack of targeted approaches for early diagnosis and treatment, the five-year survival rate of ESCC patients remains dismal [4]. The poor outcome urges development of novel preventive and therapeutic strategies against this disease, which highlights the need for better understanding of ESCC carcinogenesis.

In the past decades, studies on ESCC carcinogenesis conventionally focused on the mutation and malignant transformation of esophageal epithelium squamous cells. Notably, a few of mutated genes governing cell cycle or apoptosis (e.g. CCND1, CDKN2A, SOX2, and TP53) have been identified in a fraction of ESCC patients by comprehensive genomic characterization [5, 6]. However, these discoveries have not been well translated to the bedside and yield significant benefit for patients, partially due to the inter- and intra-tumor genomic and epigenomic heterogeneity [7]. On the other hand, accumulating results suggest that the interaction between mutant cells and immune cells in tissue microenvironment directly influences and even determines the development of cancer [8, 9]. Importantly, immunotherapies that target tumor microenvironment instead of tumor intrinsic cells have revealed remarkable efficacy in multiple cancer types, shedding light on the possible treatment of ESCC [10–12]. However, the sophisticated immune responses and their biological significance during ESCC carcinogenesis is still unclear.

In our previous study, transcriptional profiling with ESCC rat model suggested that migration and aggregation of immune cells regulated by chemokine signaling were markedly altered during esophageal carcinogenesis (Supplementary Figure S1a and b). It was worth noting that chemokine (C-C motif) ligand 2 (CCL2) as the leading chemokine was prominently over-expressed in esophageal tumors (Supplementary Figure S1c). In tumor microenvironment, CCL2 interacts with C-C motif chemokine receptor 2 (CCR2) to mediate chemotaxis of monocytes and tumor associated macrophages (TAMs), which consequently contributes to the shaping of tumor microenvironment and facilitates cancer progression [13, 14]. Though TAMs have been indicated as a promising therapeutic target to treat some cancers, our understanding on its activation and tumor-promoting mechanism is limited [15–18]. Particularly, the involvement of CCL2-CCR2 and TAMs in ESCC has not been investigated yet. In this study, we demonstrated the antitumor activity of CCL2-CCR2 blockade in esophageal carcinogenesis and deciphered the mechanism underlying tumor evasion induced by TAMs.

## Methods

### Patient cohorts

Human ESCC tissue microarray chips of two cohorts were obtained from the Shanghai Outdo Biotech Company (Shanghai, China). Cohort I contains normal mucosa (10 cases), dysplasia (22 cases), and ESCC (58 cases). Each case contains two independent tissue samples on the chip. In Cohort II, clinical samples containing tumors and matched adjacent tissues were obtained from 100 ESCC patients that enrolled from January 2009 to December 2010 and followed up for 6.5 years. The clinic pathological and follow-up data of patients were prospectively collected (Supplementary Table S1).

### Animal models

Six-week-old male F344 rats and C57BL/6 mice were purchased from Beijing Vital River Laboratory Animal Technology Company (Beijing, China). C57BL/6 CCL2^−/−^ mice (Ccl2^*tm1Rol*^/J), CCR2^−/−^ mice (Ccr2^*tm1Ifc*^/J) and PPARγ^loxP^ mice (Pparg^tm2Rev^/J) were obtained from the Jackson laboratory. PPARγ^loxP^ mice were bred with Lyz2^cre^ mice to generate macrophage-specific PPARG deletion mice (PPARG^−/−ΔMacrophage^). The mice were bred at the Shanghai Model Organisms Center (Shanghai, China) and used between the ages of 4 and 8 weeks. The ESCC rat model [19, 20] and mouse model [21] have been previously established in our lab.

### Cell lines

The immortalized human normal esophageal epithelium cell line Het-1A (CRL-2692) and human monocyte cell line THP-1 (TIB-202) was obtained from American Type Culture Collection (ATCC, VA, USA). Het-1A cells were cultured in Bronchial epithelial cell basal medium (BEGM) with all the additives (Lonza, MD, USA). The human ESCC cell line TE-1 cells were obtained from the Cell Bank of Shanghai Institutes for Biological Sciences (Chinese Academy of Sciences, Shanghai, China). THP-1 cells and TE-1 cells were cultured in RPMI-1640 medium supplemented with 10% fetal bovine serum, penicillin (100 μg/ml) and streptomycin (100 μg/ml). All the cell lines were authenticated by short tandem repeats (STR) profiling (Supplementary materials).

### TCGA data and gene set enrichment analysis (GSEA)

The provisional TCGA Esophageal Carcinoma data sets referenced during the study are available in a public repository from the Genomic Data Commons (https://portal.gdc.cancer.gov/) and from the TCGA manuscript publication page (https://www.cancer.gov/). The cases included in this study are listed in Supplementary Table S2. Gene set enrichment analysis (GSEA) was performed using GSEA software and Molecular Signatures Database (MSigDB) gene sets downloaded from Broad Institute (http://software.broadinstitute.org/gsea/index.jsp). RNA sequencing data is available through the National Center for Biotechnology Information Gene Expression Omnibus (NCBI–GEO) database (http://www.ncbi.nlm.nih.gov/geo/) under the accession number GSE134067.

### Statistical analysis

Statistical analysis was conducted using GraphPad Prism software. All data represents at least three independent experiments and are expressed as mean ± standard deviation. Log-rank test and multi-variate COX were used to estimate patients’ overall survival. Two-way analysis of variance (ANOVA) or one-way ANOVA followed by Bonferroni’s test was used for multiple groups’ analysis. Unpaired Student’s t-test was used to determine statistical significance in two-group experiments. *P* value less than 0.05 was considered statistically significant.

Other detailed information on materials can be found in the Supplementary methods, Supplementary Figure S2, and Supplementary Tables S3-S4.

## Results

### CCL2 and TAMs correlate with esophageal carcinogenesis and predicts poor prognosis in ESCC patients

**Fig. 1:**
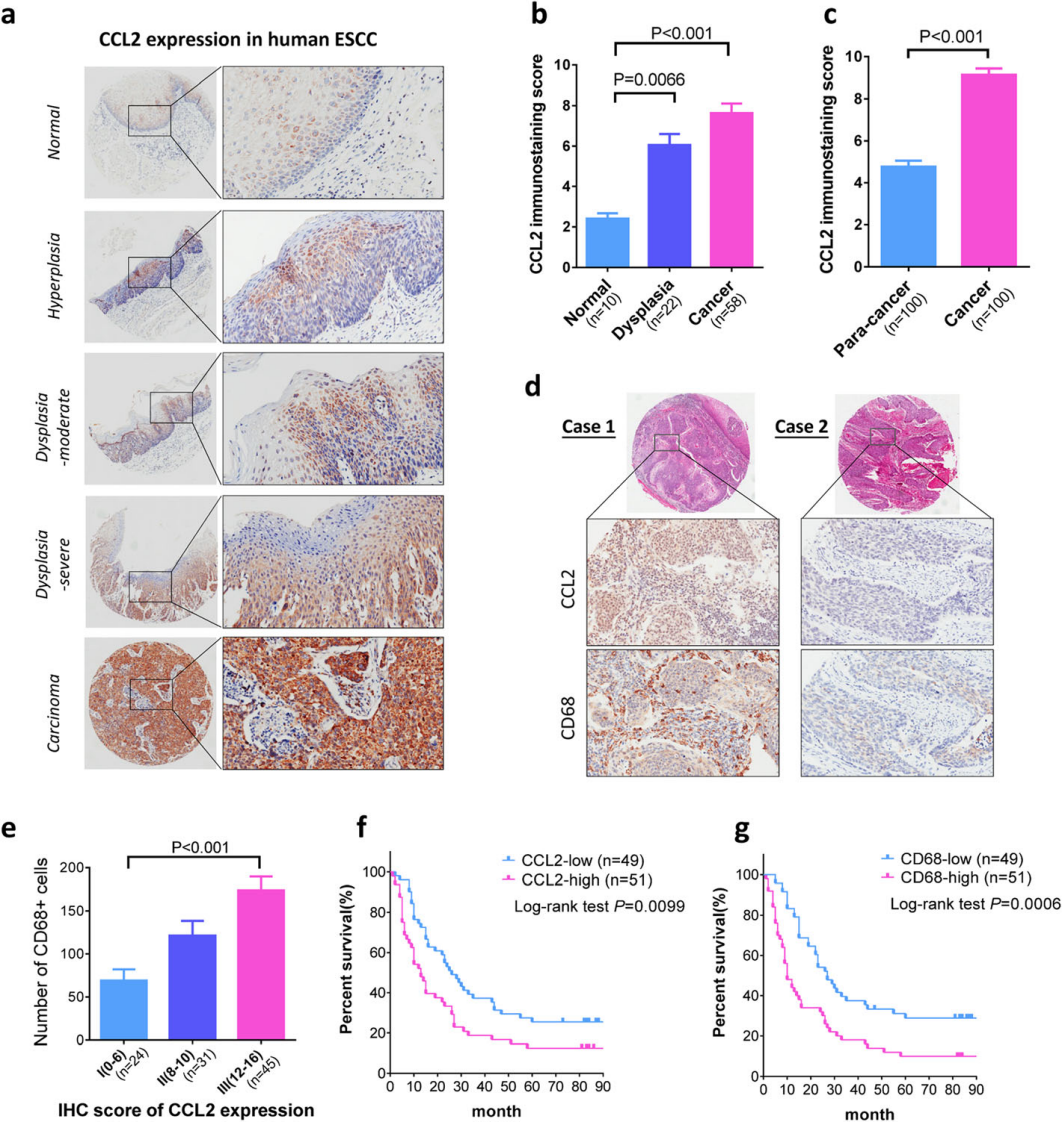
CCL2 expression correlates with TAMs accumulation, cancer progression and poor prognosis in human ESCC. **a** Representative IHC staining indicates escalating expression of CCL2 with histopathologic progression. **b** Expression of CCL2 in different pathologic grades of cohort I patients including normal mucosa (10 cases), dysplasia (22 cases), and ESCC (58 cases). **c** Expression of CCL2 in para-cancer and cancer tissues of cohort II patients (*n* = 100). **d** Representative IHC staining indicates correlated expression of CCL2 and CD68 in ESCC. **e** CCL2 expression is correlated with accumulation of CD68^+^ TAMs. **f** and **g** High-expression of CCL2 (**f**) and CD68 (**g**) predicts reduced overall survival of ESCC patients

To evaluate the association of CCL2 and TAMs in esophageal carcinogenesis, we firstly determined the distribution of CCL2 with human tumor tissue microarrays constructed from ESCC patients (cohort I). The expression level of CCL2 was low in normal mucosa and hyperplasia, but continuously increased in the progression of pathological lesions including dysplasia, papilloma and carcinoma (Fig. 1a and b). In another cohort, the expression of CCL2 was markedly enhanced in cancer tissues when compared to the paired para-cancer tissues (Fig. 1c). Next, we compared CCL2 expression with the number of cells expressing CD68, which is a common marker for TAMs. In ESCC cases with higher expression of CCL2, the number of TAMs was significantly elevated (Fig. 1d and e). Collectively, these data confirmed the tight association of CCL2 and TAMs with carcinogenesis in human ESCC. In addition, the correlation between expression of CCL2 and CD68 with the overall survival of patients was investigated. In the cohort followed up for 4.6 to 6.5 years, expressions of both CCL2 and CD68 were inversely associated with the overall survival of ESCC patients (Fig. 1f and g). Multivariate Cox analysis suggested that CCL2 expression was an independent prognosticator of overall survival for human ESCC (*P* = 0.013, Supplementary Table S5).

### CCL2 correlates with TAMs accumulation and tissue inflammation in nitrosamine-induced esophageal carcinogenesis

**Fig. 2:**
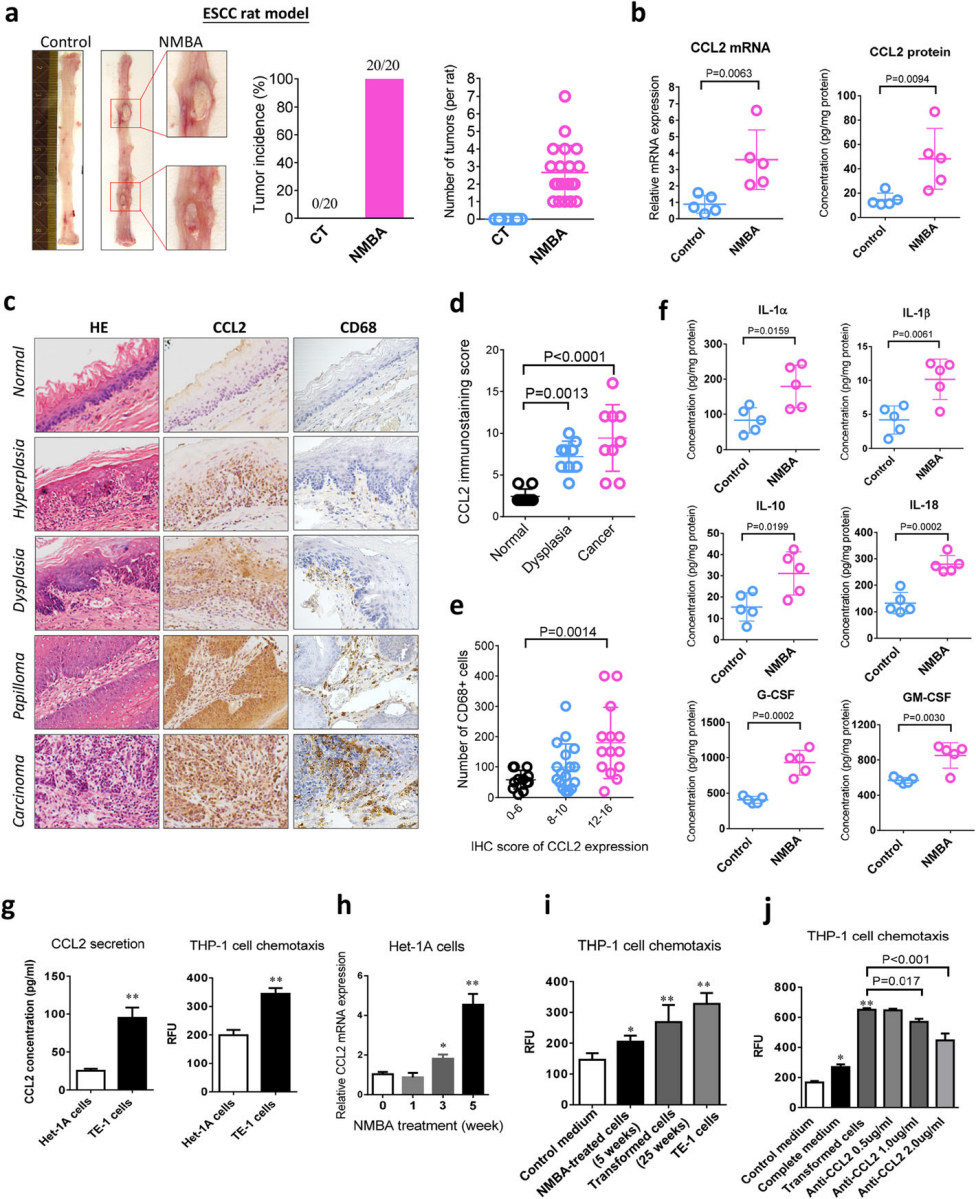
CCL2 correlates with TAMs accumulation and tissue inflammation in nitrosamine-induced esophageal carcinogenesis. **a** NMBA induces notable tumors in ESCC rat model. **b** Expression of CCL2 in rat esophageal epithelium is increased at mRNA and protein levels during carcinogenesis (*n* = 5). **c** Representative IHC staining indicates the expression of CCL2 and CD68 during rat esophageal carcinogenesis resembling human ESCC. **d** Expression of CCL2 increases with pathologic progression in rat model (*n* = 9). **e** Accumulation of CD68^+^ TAMs is correlated with CCL2 expression. **f** Increased expression of inflammatory cytokines during esophageal carcinogenesis (n = 5). **g** The basal levels of CCL2 between normal human esophageal epithelium cells (Het-1A) and the ESCC cells (TE-1). **h** NMBA treatment continuously increases CCL2 expression over time. **i** Chemotaxis of THP-1monocyte is increased by conditioned medium from the transformed cells and TE-1 cells. **j** Monocyte chemotaxis induced by transformed cells is antagonized by CCL2-neutralizing antibody in a dose-dependent manner. Data is shown by mean ± standard deviation from three independent experiments. When compared to the control group, * indicates *P* < 0.05, ** indicates *P* < 0.01

To validate the effect of CCL2 on TAMs infiltration during esophageal carcinogenesis, we determined the tissue levels of CCL2 and CD68 with the ESCC rat model (Fig. 2a). In this animal model, NMBA-treatment (15 injections in 5 weeks) specifically induced noticeable tumors in rat esophagus after 35 weeks, with 100% tumor incidence and nearly 3 visible tumors per rat (Fig. 2a). Compared to the animals of vehicle control, the mRNA and protein levels of CCL2 were significantly increased in the esophageal epithelium of NMBA-treated rats (Fig. 2b). Consistent with our finding from human ESCC cohorts, the expression of CCL2 and the number of CD68 positive macrophages were remarkably elevated in concordance with the pathological progression of rat esophageal epithelium (Fig. 2c, d and e). Additionally, the inflammatory cytokines in esophageal tissue including IL-1α/β, IL-10, IL-18, G-CSF and GM-CSF were significantly increased during the development of tumors (Fig. 2f). Thus, our data from ESCC cohorts and rat model both suggested that escalated expression of CCL2 and TAMs accumulation play an important role in esophageal carcinogenesis.

Next, we further studied the interaction between CCL2 and TAMs with human-origin cell models in vitro. Firstly, we compared the basal levels of CCL2 expression between normal human esophageal epithelial cells (Het-1A) and the ESCC cells (TE-1). In comparison with Het-1A cells, CCL2 secretion in culture medium was markedly higher in TE-1 cancer cells, and the chemotaxis assay with human monocyte THP-1 cells indicated that monocyte migration was also increased in TE-1 conditioned medium (Fig. 2g). To investigate how tumor cells affecting TAMs during ESCC carcinogenesis, we constructed a malignant transformation cell model with continuous NMBA-treatment in Het-1A cells (Supplementary Figure S3a). The NMBA treatment continuously elevated CCL2 expression over time until the malignant transformation was successfully induced at Week 25 (Fig. 2h). In parallel with CCL2 expression, chemotaxis of THP-1 cells was also increased by the conditioned medium from malignantly transformed cells, which was comparable to the effect of TE-1 cells (Fig. 2i). In addition, monocyte chemotaxis induced by transformed cells was significantly antagonized by CCL2-neutralizing antibody in a dose-dependent manner (Fig. 2j). These data further validated our in vivo observations regarding that CCL2 expression triggered TAMs infiltration during esophageal carcinogenesis.

### Blockade of CCL2-CCR2 axis suppresses monocyte infiltration, TAMs accumulation and tumorigenesis

**Fig. 3:**
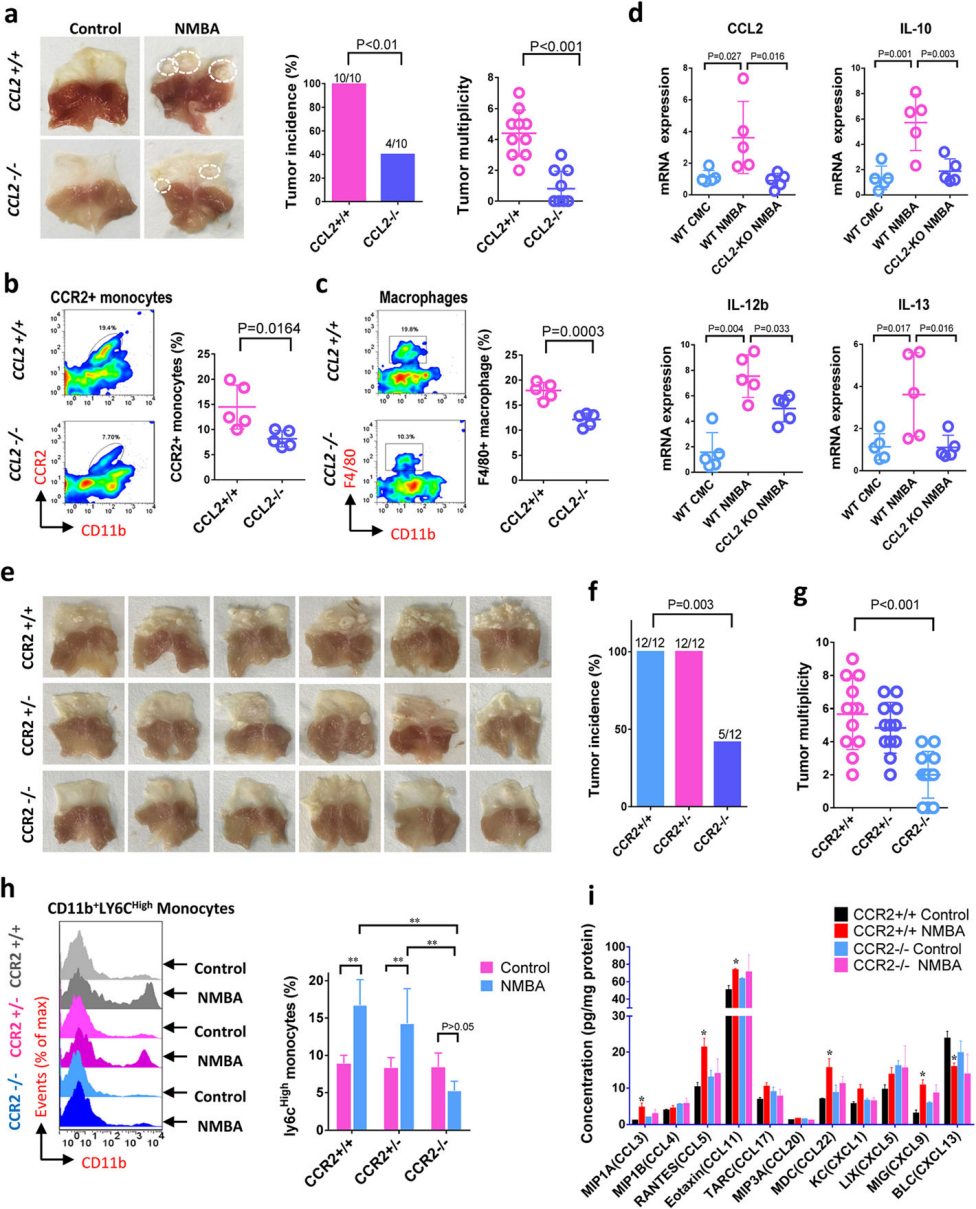
Blockade of CCL2-CCR2 axis suppresses monocyte infiltration, TAMs accumulation and tumorigenesis in ESCC mouse model. **a** Gene knockout of CCL2 in mouse reduces tumor incidence and multiplicity (n = 10). **b** Deletion of CCL2 in mouse suppresses infiltration of CD11b^+^CCR2^+^ monocyte and CD11b^+^F4/80^+^ TAMs in forestomach tumors (*n* = 5). **c** Deletion of CCL2 in mouse inhibited production of TAMs-associated cytokines (CCL2, IL-10, IL-12b, and IL-13) (n = 5). **d**, **e** and **f** Gene knockout of CCR2 reduces tumor incidence (**e**) and tumor numbers (**f**) in mouse forestomach (*n* = 12). **g** Infiltration of CD11b^+^Ly6C^high^ inflammatory monocyte elevated in CCR2^+/+^ wild type mice and CCR2^+/−^ heterozygous mice is blocked in CCR2^−/−^ animals (*n* = 6). **h** CCR2 knockout suppresses production of inflammatory cytokines in tumors (n = 6). Data is shown by mean ± standard deviation, * indicates *P* < 0.05, ** indicates *P* < 0.01, when compared to the CCR2^+/+^ control

To further explore the function of tumor infiltrating macrophages in esophageal carcinogenesis, we constructed ESCC mouse model with CCL2 and CCR2 gene deletion. Similarly to the ESCC rat model, continuous increase of CCL2 expression and TAMs accumulation were also observed in the forestomach of wild type mouse during carcinogenesis (Supplementary Figure S4a). More importantly, gene knockout of CCL2 dramatically decreased the incidence and number of forestomach tumors in the mouse model (Fig. 3a), suggesting the crucial role of CCL2 in the development of ESCC. Furthermore, we found that deletion of CCL2 significantly inhibited the infiltration of CD11b^+^CCR2^+^ monocyte (Fig. 3b) as well as the accumulation of CD11b^+^F4/80^+^ TAMs (Fig. 3c) in tumors. In addition, CCL2 knockout induced marked suppression on TAMs-associated inflammatory cytokines such as IL-10, IL-12b, and IL-13, which were prominently elevated by NMBA-treatment in wild type animals (Fig. 3d).

As the key functional receptor for CCL2, CCR2 is expressed primarily on the cell surface of monocyte and TAMs. Hence, we further conducted ESCC carcinogenesis study using the CCR2^−/−^ mouse (Fig. 3e). In line with our findings with CCL2^−/−^ mouse model, the loss of CCR2 markedly reduced the incidence of mouse forestomach tumors by nearly 60%, as compared with those in CCR2^+/+^ wild type and CCR2^+/−^ heterozygous animals (Fig. 3f). The average number of tumors declined from 5.7 to 2.0 per mouse (Fig. 3g). Flow cytometry analysis showed that CD11b^+^Ly6C^high^ inflammatory monocytes were notably infiltrated in forestomach tumors of CCR2^+/+^ and CCR2^+/−^ mice after NMBA treatment; however, the infiltration was completely abolished in CCR2^−/−^ mice (Fig. 3h). Furthermore, profiling of tissue cytokines indicated noticeable suppression of inflammatory chemokines including MIP-1α (CCL3), RANTES (CCL5), Eotoxin (CCL11), MDC (CCL22), and MIG (CXCL9) by the deletion of CCR2 (Fig. 3i), suggesting that the inflammatory responses prompted by TAMs accumulation was equilibrated by the blockade of CCL2-CCR2 axis.

### TAMs mediate tumor cell evasion through programmed death-1 (PD-1) signaling pathway

**Fig. 4:**
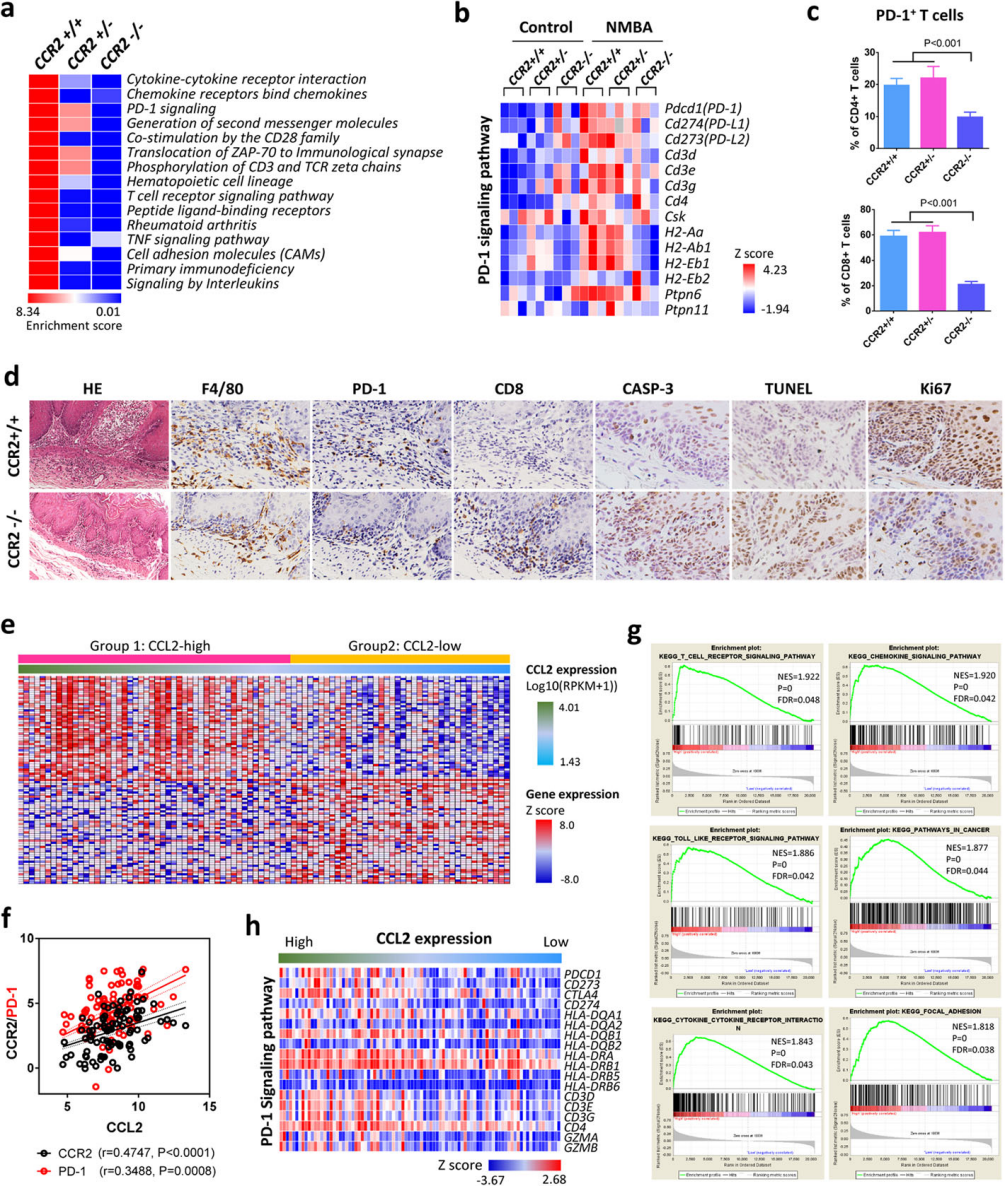
TAMs mediate immune evasion through PD-1 signaling pathway. **a** Heatmap of enriched pathways (Top 15) from differentially expressed genes from CCR2^+/+^ wild type mice, CCR2^+/−^ heterozygous mice, and CCR2^−/−^ animals. **b** Heatmap shows the expression of genes that associated with PD-1 signaling pathway is activated in carcinogenesis but repressed by CCR2 knockout. **c** Flow cytometry analysis demonstrates suppressed PD-1 expression in CD4^+^ and CD8^+^ T cells by CCR2 deletion (n = 6). **d** Representative IHC staining of F4/80, PD-1, CD8, cleaved CASP-3, Ki67 and TdT-mediated dUTP nick end labeling (TUNEL) in tumor microenvironment of CCR2 knockout ESCC mouse model. **e** Heatmap of gene expression profiles of human ESCC cases (*n* = 90) from TCGA database. **f** Pearson correlation analysis shows tight association of CCL2 with CCR2 and PD-1. **g** Gene set enrichment analysis (GSEA) between group high (*n* = 52) and low (*n* = 38) of CCL2 expression shows enriched pathways associated with PD-1 signaling. **h** Heatmap shows that expression of PD-1 signaling pathway associated genes is correlated with CCL2 expression in human ESCC

To explicate the mechanism underlying tumor promotive function of infiltrated TAMs, we carried out RNA-sequencing with tumors harvested from the CCR2^−/−^ mouse model (Supplementary Figure S4b). In parallel with our previous findings from ESCC rat model (Supplementary Figure S1), gene expression changes during carcinogenesis were remarkably enriched in the pathways interconnected with immune responses, which were led by the pathway of “cytokine-cytokine receptor interaction” and “chemokine receptors bind chemokines”. Moreover, “PD-1 signaling” pathway that negatively controls T cells activities was identified to be most significantly suppressed by CCR2 knockout (Fig. 4a). In addition, the expression of genes specifically involved in PD-1 pathway was activated in NMBA-induced carcinogenesis, but notably repressed by the deletion of CCR2 (Fig. 4b). In concert with this, other pathways associated with PD-1 signaling including “CD28 family”, “ZAP-70”, “Phosphorylation of CD3” and “TCR signaling” were similarly constrained by the absence of CCR2.

To validate the inhibitory effect of CCR2 blockade on PD-1 signaling, we further analyzed the expression of PD-1 in T cells using CCR2^−/−^ mouse model. Flow cytometry analysis showed that the proportion of PD-1^+^ T cells in CD8^+^ CTLs was decreased from nearly 60% down to 20% by CCR2 deletion (Fig. 4c). Similarly, PD-1 expression in CD4^+^ cells of CCR2^−/−^ mice was also significantly decreased. In addition, to better elucidate the correlation between TAMs and CTLs, we performed immunostaining of F4/80, PD-1 and CD8 in the tumors. In comparison with the CCR2^+/+^ animals, the numbers of F4/80^+^ TAMs and PD-1^+^ T cells in tumor microenvironment were markedly decreased in CCR2^−/−^ mice. In contrast, distribution of CD8^+^ CTLs was notably recuperated by the knockout of CCR2 (Fig. 4d). Immunostaining of cleaved CASP-3 and TdT-mediated dUTP nick end labeling (TUNEL) indicated the activation of pro-death signaling in tumor cells, while Ki67 staining verified tumor suppression in CCR2^−/−^ mice (Fig. 4d). Together, the inverse correlation of CD8^+^ CTLs with CCR2 and PD-1 suggested that TAMs mediate depletion of antitumor T cells and in consequence facilitates tumor cell evasion through PD-1 signaling pathway.

### Activation of PD-1 signaling is closely corelated with CCL2-CCR2 axis in human ESCC

To confirm the connection between CCL2-CCR2 and PD-1 in human esophageal carcinogenesis, we next performed the gene set enrichment analysis (GSEA) using ESCC expression profiles from TCGA database (Fig. 4e). Firstly, it was confirmed that CCL2 expression was significantly correlated with the levels of CCR2 and PD-1 in the 90 ESCC cases (Fig. 4f). Next, GSEA indicated that six KEGG pathways were significantly (with a stringent cutoff for FDR 5% and *p* value 0.01) enriched in the “High” expression group of CCL2, including “T cell receptor signaling”, “chemokines signaling”, “Toll-like receptor signaling”, “pathways in cancer”, “cytokine-cytokine receptor interaction”, and “focal adhesion” (Fig. 4g, Supplementary Table S6). This is in line with our observations with animal models, which suggest the prominent involvement of immune process in ESCC carcinogenesis (Fig. 4a, Supplementary Figure S1). Particularly, the expression of genes implicated in PD-1 signaling pathway was closely correlated with CCL2 expression in ESCC patients (Fig. 4h). Thus, TCGA data also indicates the tight connection between CCL2-CCR2 axis and PD-1 signaling. Taken together, these data strongly suggest that CCL2-CCR2 axis promotes carcinogenesis by inducing TAMs-mediated immune escape via PD-1 signaling pathway.

### Depletion of antitumor effector T cells is associated with M2-polorization of TAMs in ESCC carcinogenesis

**Fig. 5:**
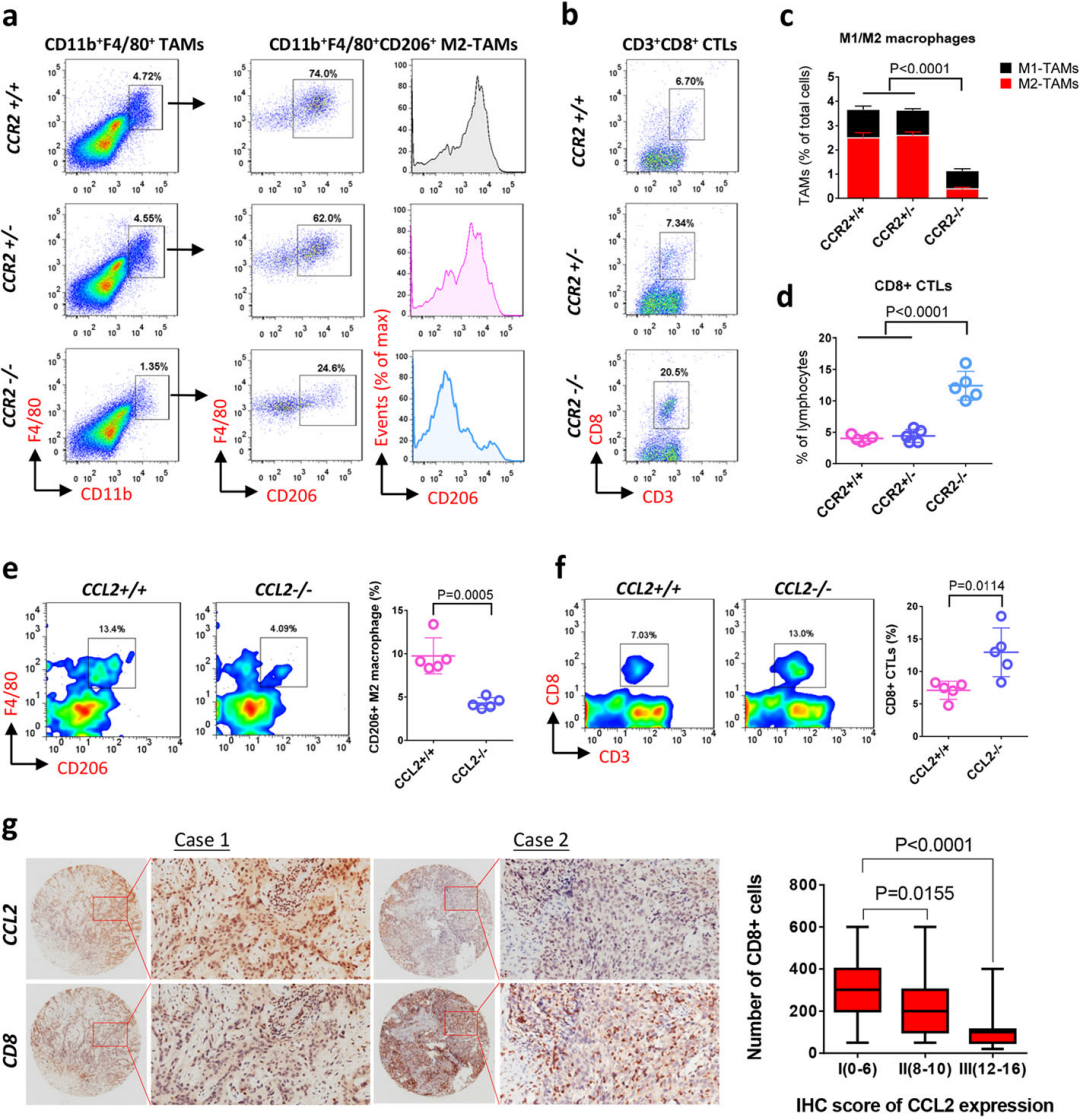
M2-polorization of TAMs is associated with depletion of antitumor effector T cells in ESCC carcinogenesis. **a** and **c**) Flow cytometry shows inhibition of TAMs (CD11b^+^F4/80^+^) accumulation as well as M2 polarization (F4/80^+^CD206^+^) by CCR2 knockout. Data is shown by mean ± standard deviation (n = 6). **b** and **d** CCR2 knockout increases antitumor effector T cells (CD3^+^CD8^+^ CTLs) in tumors (n = 6). **e** and **f** CCL2 deletion inhibits TAMs M2-polorization (**e**) and antitumor effector T cells depletion (**f**) during carcinogenesis (n = 5). **g** Representative IHC staining indicates expression of CCL2 inversely correlated with CD8 (effector T cells) in human ESCC cohort II (*n* = 100)

Besides TAMs accumulation, the differentiation of macrophages in tumor microenvironment generates particular TAMs subtypes with diverse functional properties, thereby impacting the development of cancer. In this study, we observed that the activation state of TAMs in ESCC mouse model (CCR2^+/+^ wild type) was predominantly M2-type (over 70%), as indicated by the proportion of CD206^+^ in CD11b^+^F4/80^+^ macrophages (Fig. 5a). In addition to the decrease of TAMs accumulation by CCR2 knockout, M2-polorization was also dramatically inhibited in the CCR2^−/−^ animals by nearly 70% when compared to the CCR2^+/+^ and CCR2^+/−^ mice (Fig. 5a and c). Interestingly, we found that the proportion of CD8^+^ cytotoxic T cells (CTLs) in CD3^+^ lymphocytes was significantly elevated by the deletion of CCR2 (Fig. 5b and d). Similar results were also observed in the CCL2 knockout mouse model, in which F4/80^+^CD206^+^ M2-type of TAMs in total CD45^+^ cells was decreased by over 50% (Fig. 5e), while the number of CD8^+^ CTLs in tumors was inversely increased up to 2-fold of that in CCL2^+/+^ group (Fig. 5f). To further validate the impact of CCL2 on CTLs depletion, we detected the distribution of CCL2 and CD8 in tumor tissues with ESCC patients (cohort II). The immunohistochemistry (IHC) staining showed that higher expression of CCL2 was significantly correlated with decreased number of CD8 positive antitumor T cells (Fig. 5g). Taken together, these data suggested that M2-polorization of infiltrated TAMs facilitates the depletion of antitumor effector T cells in tumor microenvironment.

### M2 polarization of TAMs facilitate immunosuppression through elevating the expression of PD-L2

**Fig. 6:**
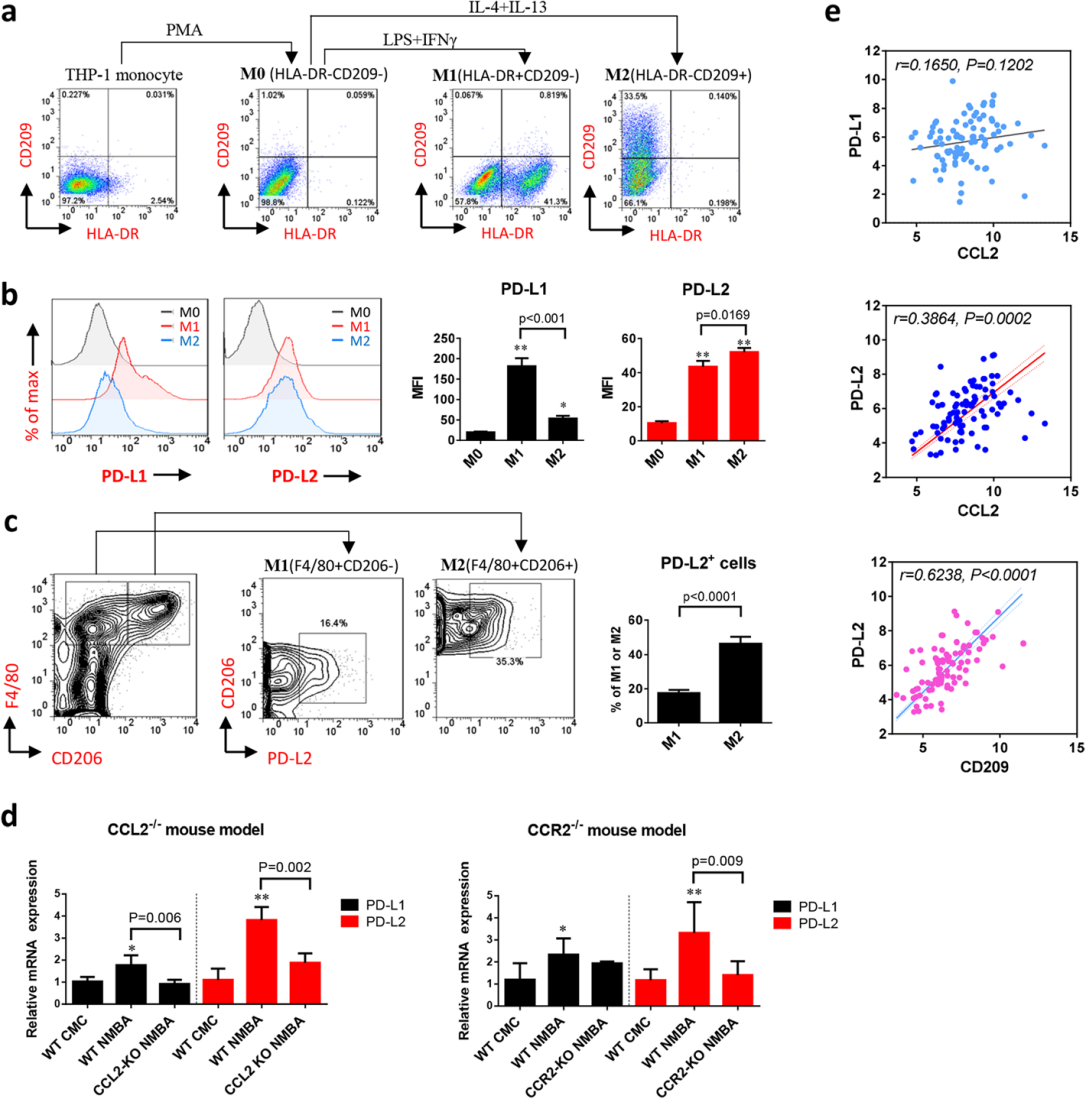
M2 polarization of TAMs facilitates immunosuppression through the elevated expression of PD-L2. **a** THP-1 monocyte is induced into M1-type by LPS and IFN-γ, and into M2-type by IL-4 and IL-13 following PMA treatment. The polarized M1 and M2 macrophages were distinguished from inactive M0 macrophage with the biomarker HLA-DR and CD209, respectively. **b** Flow cytometry analysis shows discrepant expression of PD-L1 and PD-L2 between HLA-DR^+^CD209^−^ M1 macrophages and HLA-DR^−^CD209^+^ M2 macrophages. Data is shown by mean ± standard deviation from three independent experiments. When compared to the control group, * indicates P < 0.05, ** indicates *P* < 0.01. **c** PD-L2 expression is higher in M2 (F4/80^+^CD206^+^) than M1 (F4/80^+^CD206^−^) macrophages in ESCC mouse model (*n* = 5). **d** Blockade of CCL2-CCR2 axis in animal models reduces expression of PD-L2 during carcinogenesis (*n* = 5). **e** Pearson correlation analysis with TCGA data indicates strong association of PD-L2 with CCL2 expression and the level of M2 marker CD209 in human ESCC

To interpret the impact of TAMs polarization on PD-1 signaling, we next determined the expression of two specific ligands of PD-1 that responsible for T cells exhaustion, PD-L1 and PD-L2, in macrophages at particular activation states. In our cell model, THP-1 cells were induced into macrophages of M1-type by LPS and IFN-γ, and into M2-type by IL-4 and IL-13 following PMA treatment. The polarized M1 and M2 macrophages were distinguished from inactive M0 macrophage with the biomarker HLA-DR and CD209, respectively (Fig. 6a). Interestingly, PD-L1 was highly expressed in the HLA-DR^+^CD209^−^ M1 macrophages, but not prominently expressed in the HLA-DR^−^CD209^+^ M2 macrophages (Supplementary Figure S6a). In contrast, PD-L2 was particularly expressed in the M2-type, which is higher than that in M1 macrophages (Fig. 6b). Similarly, PD-L2 expression in F4/80^+^CD206^+^ M2 TAMs was significantly higher than M1 TAMs in the ESCC mouse model (Fig. 6c). In wild type mouse, both PD-L1 and PD-L2 were increased by NMBA-induced carcinogenesis, but the over-expression of PD-L2 appeared more pronounced than PD-L1 (Fig. 6d). More importantly, blockade of CCL2 or CCR2 dramatically suppressed the expression of PD-L2 in tumors (Fig. 6d). Together with our finding that M2 was the prevailing subtype of TAMs in ESCC mouse model (Fig. 5a and c), this highlights the role of TAMs-specific PD-L2 in esophageal carcinogenesis. Consistent with cell models and mouse models, TCGA data also demonstrated the strong correlation of PD-L2 with CCL2 expression as well as the level of M2 marker CD209 in ESCC patients; in contrast, PD-L1 was not significantly correlated (Fig. 6e).

**Fig. 7:**
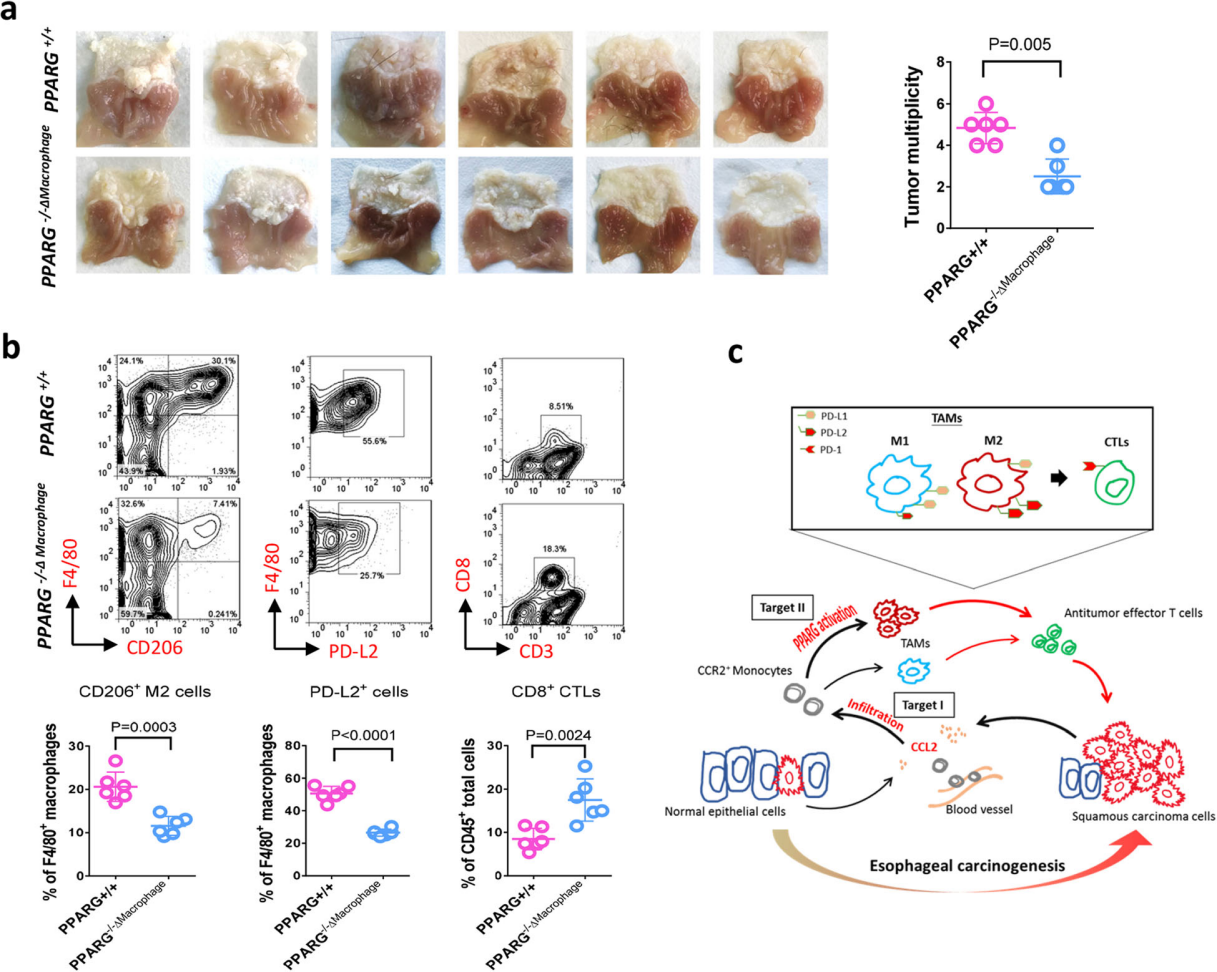
Blockade of TAMs M2-polarization via macrophage-specific PPARG deletion reduces PD-L2 expression and inhibits tumorigenesis. **a** Macrophage-specific gene knockout of PPARG in mouse reduces tumor growth in ESCC mouse model (n = 6). **b** Flow cytometry analysis shows deficiency of PPARG in macrophage suppresses M2-polarization and PD-L2 expression in TAMs and increases CD8+ CTLs in forestomach tumors (n = 6). **c** Schematic figure indicates esophageal carcinogenesis can be blocked by targeting TAMs infiltration via CCL2/CCR2 signaling (Target I) and M2 polarization via PPARG activation (Target II)

To further validate the impact of M2-polarization, we next conducted esophageal carcinogenesis study using mice with macrophage-specific deletion of peroxisome proliferator activated receptor-γ (PPARG) which has been shown required for the maturation of M2 macrophages [22, 23]. The results indicated that macrophage-specific PPARG deletion significantly inhibited tumorigenesis in ESCC mouse model (Fig. 7a). The blockade of M2 polarization by PPARG deficiency dramatically decreased expression of PD-L2 in TAMs, but inversely increased the CD8^+^ antitumor effector T cells in tumors (Fig. 7b). In summary, these data are in line with our findings on the interaction between TAMs-M2 polarization and PD-1 signaling activation, suggesting that the presentation of PD-L2 by M2-TAMs constitutes an important mechanism underlying immune evasion in ESCC carcinogenesis.

## Discussion

It has been recognized that the development of cancer is influenced by interactions between tumor cells and host immune response [24]. Within tumor microenvironment, TAMs contribute substantially to inflammatory homeostasis and in consequence impacts cancer progression [25, 26]. The functions of TAMs have been characterized in various cancer types, but little is known about the mechanism of action in esophageal cancer. Here, we show the pivotal role of TAMs in esophageal carcinogenesis by targeting infiltration and polarization (Fig. 7c). We found that tumorigenesis was remarkably suppressed by the blockade of CCL2-CCR2 axis in ESCC animal models. Mechanistically, this can be attributed to the inhibition of TAMs recruitment and M2 polarization, thereby halting the immunosuppression on antitumor effector T cells through PD-1 signaling pathway. Since TAMs-targeted therapeutics are being clinically evaluated as a promising option for some cancer types [27], our findings harbor direct translational impact for the prevention and treatment of human ESCC.

The accumulation of TAMs in microenvironment is due to self-proliferation and recruitment from circulating inflammatory Ly6C^+^CCR2^+^ monocytes [25]. The latter is primarily mediated by elevated secretion of the monocyte chemoattractant protein MCP-1/CCL2 by tumor cells [14, 28]. Increasing studies indicated that inhibition of CCL2 could deplete inflammatory monocytes and macrophages, reduce tumor growth and dissemination in different experimental models such as prostate, melanoma, breast, lung and liver cancer [29–32]. In regard to ESCC, early studies had shown the correlation of increased CCL2 expression with macrophage infiltration and tumor invasion [33, 34]. Paralleling previous reports, our study demonstrated that CCL2 acts to bridge tumor cells with TAMs-associated immune response rather than directly impact ESCC markers (Supplementary Figure S5) in the tissue microenvironment during esophageal carcinogenesis.

The upstream molecular mechanism governing CCL2 expression in pre-malignant or malignant epithelial cells remains to be interpreted. Our microarray profiling with human esophageal cell (Het-1A) model suggested that continuous elevation of CCL2 during malignant transformation might be due to the activation of PI3K-AKT signaling pathway (Supplementary Figure S3b, c, and d). However, chemotherapeutic approaches usually result in limited clinical efficacy when aiming at tumor intrinsic signaling pathways [35, 36], primarily due to heterogeneity in tumors and unexpected impacts on the favorable immune cells in ESCC microenvironment [37]. In contrast, TAMs-centered therapeutic strategies have demonstrated remarkable potential to complement and synergize with chemotherapy and immunotherapy [38, 39]. As such, novel strategies targeting CCL2-CCR2 or TAMs might be promising options for ESCC, provided that the mechanism of action is elucidated.

The differentiation of TAMs is directed by cytokines in tumor microenvironment. IL-4 and IL-13 are major drivers of M2 polarization, as is also validated by the THP-1 cell model and mouse models in our study. It is worth noting that monocyte chemotaxis was successfully induced by tumor-educated mediums but M2 polarization was not clearly observed in the same condition (Supplementary Figure S6b), suggesting that other non-cancer cells e.g. Th2 cells and cancer-associated fibroblasts (CAFs) are requisite for TAMs differentiation [40]. Tumor promotive activity of M2-type TAMs has been observed in numerous studies. However, the underlying mechanism has not been clearly explained. It was reported that M2 phenotype macrophages infiltrated following infiltration of M1 macrophage and promoted esophageal carcinogenesis in a surgical rat model [41]. In tumor biopsies of patients, reduced CD163^+^ M2-TAMs was inversely correlated with the increased CD8^+^/CD4^+^ T cells ratio [42]. Consistent with these studies, our data demonstrated that M2 polarization of TAMs was tightly associated with reduced CD8^+^ T cells and promoted tumor growth. Furthermore, multiple evidences in this study suggested that PD-1 signaling pathway played a crucial role in TAMs-mediated immune evasion during esophageal carcinogenesis.

Engagement of PD-1with its two ligands PD-L1and PD-L2 is responsible for tumor escape through defeating the antitumor capacity of tumor-specific CTLs. Immune checkpoint blockade targeting PD-1/PD-L1 has been proven effective for cancer treatment via the paradigm of “immune normalization” [43]. Despite FDA approval of this strategy for multiple cancer types, the underlying mechanism for T cells regulation in microenvironment is not yet fully understood. Tumoral PD-L1 expression has been shown to be a predictive marker for response to anti-PD-1 targeted therapies. However, some PD-L1-positive patients of esophageal cancer did not benefit from such therapies, while some patients lack of PD-L1 still showed clinical response [10, 12], implying that other molecular interacts with PD-1 such as PD-L2 may be important for immunotherapy efficacy in ESCC. In prostate cancer, PD-L2 was more highly expressed than PD-L1 and overwhelmingly correlated with immune-related pathways, suggesting the critical role of PD-L2 in immune response [44]. Nonetheless, PD-L2 expression was independently associated with clinical response in pembrolizumab-treated (PD-1 mAb) patients with colorectal cancer, indicating that PD-L2 may be involved in response to PD-1 axis targeted therapies [45]. Although antagonist PD-1 mAb could block both PD-L1 and PD-L2 binding to PD-1, better outcomes were observed for PD-L2-positive than PD-L2-negative patients in head and neck squamous cell carcinomas treated with pembrolizumab [46]. Therefore, therapies targeting PD-L2/PD-1 interaction may provide notable clinical benefit for these cancers. However, studies that assess the prevalence of PD-L2 and especially the distribution in human tumors are limited. On the other hand, PD-L2 and PD-L1 could be differentially expressed by tumor cells or macrophages [47]. Furthermore, we observed discrepant expression of PD-L2 in M1 and M2 macrophages in our animal and cell study. Importantly, this discrepancy is largely responsible for the tumor promotion induced by the M2-type TAMs in esophageal carcinogenesis. Thus, molecules controlling M2-polarization of TAMs e.g. PPARG activation may serve as promising novel immune checkpoint target for ESCC (Fig. 7c).

## Conclusions

In summary, our study highlights the role of CCL2-CCR2 axis in esophageal carcinogenesis. Blockade of CCL2-CCR2 axis strongly suppressed cancer development through inhibiting monocyte infiltration and TAMs accumulation in tumor microenvironment. Importantly, TAMs polarization to the immunosuppressive M2 type significantly increased expression of PD-L2 and consequently depleted antitumor effector T cells. These findings provide new insights into the mechanism of immune evasion mediated by TAMs in ESCC, which may advance the development of macrophages-based strategies for ESCC prevention and immunotherapy.

## Supplementary information

**Additional file 1: Supplementary Figure S1.** Transcriptomic analysis on the NMBA-induced carcinogenesis in ESCC rat model. (a) Enrichment of Gene. Ontology (GO). (b) Enrichment of signaling pathway. The Top 30 enriched terms are shown. (c). Heatmap of chemokines and receptors in carcinogenesis. Microarray data was available on Gene Expression Omnibus (GEO) database under the accession number GSE90464. **Supplementary Figure S2.** Gating strategy in flow cytometry analysis for tumors. The antibodies used for flow cytometry has been list in Supplementary Table S4. **Supplementary Figure S3.** Escalating expression of CCL2 in the NMBA-induced ESCC carcinogenesis cell model. (a) Establishment of malignant transformation cell model with NMBA-treatment (10 μM, 25 weeks) in Het-1A cells. (b) Genes expression with the pattern of Continuous increasing over transformation. (c) Enriched pathways for the over-expressed genes. (d) Heatmap shows increased expression of CCL2 and correlated genes. **Supplementary Figure S4.** ESCC mouse model. (a) CCL2 expression and TAMs accumulation was continuously increase in mouse forestomach during carcinogenesis. (b) Heatmap of Gene expressions by RNA-sequencing with tumors harvested from the CCR2−/− mouse model. **Supplementary Figure S5.** The effect of CCL2 on pro-death signaling, proliferation and ESCC markers. (a) Heatmap showing relative expression of ESCC markers in normal and ESCC tissues of animal models. (b) The expression of EGFR, BRCA1, CCND1, Myc, Met, TP63, and CD44 were determined with q-PCR in CCL2−/− mouse model. (c) TE-1 cells were treated with CCL2 (0, 10, 50, 500 ng/ml) for 24 h and gene expression was determined by q-PCR. **Supplementary Figure S6.** Expression of PD-L1 and PD-L2 in polarized macrophages. (a) Differential expression of PD-L1 and PD-L2 in HLA-DR + CD209- M1 macrophages and HLA-DR-CD209+ M2 macrophages. (b) Polarization was not induced by culture with conditioned medium neither by co-culture with cells. THP-1 cells were treated with PMA to induce M0 macrophages as described in Supplementary materials and methods, then incubated with conditioned medium or co-cultured with indicated cells for 72 h. Cells were harvested for analysis with flow cytometry.

**Additional file 2.** Supplementary materials. Authentication of cell lines.

**Additional file 3.** Supplementary methods.

**Additional file 4:** **Supplementary Table S1.** Samples list in the ESCC cohort (100 cases). **Supplementary Table S2.** ESCC samples list from TCGA database. **Supplementary Table S3.** Mouse (m) and Human (h) Primers for PCR. **Supplementary materials S4.** Reagents and antibodies. **Supplementary table S5.** Uni- and multi-variate COX proportional hazard model analysis for overall survival of ESCC patients in cohort II. **Supplementary table S6.** KEGG pathway enrichment by GSEA analysis.

## Data Availability

All data generated or analyzed during this study are included in this published article and its supplementary information files.

## Abbreviations

CCL2: Chemokine (C-C motif) ligand 2
CCR2: C-C Motif Chemokine Receptor 2
EAC: Esophageal adenocarcinoma
ESCC: Esophageal squamous cell carcinoma
GSEA: Gene set enrichment analysis
HE: Haematoxylin and eosin
IHC: Immunohistochemistry
IL-13: Interleukin-13
IL-4: Interleukin-4
NMBA: N-nitrosomethylbenzylamine
PD-1: Programmed death-1
PPARG: Peroxisome proliferator activated receptor-γ
TAMs: Tumor associated macrophages
TCGA: The cancer genome atlas
TMA: Tumor tissue microarray
