# Supplementary material for: CCL2-CCR2 axis recruits tumor associated macrophages to induce immune evasion through PD-1 signaling in esophageal carcinogenesis
Source: Mol Cancer. 2020 Feb 27;19:41. doi: 10.1186/s12943-020-01165-x (PMC7045401; doi:10.1186/s12943-020-01165-x)
Supplement: Supplementary file 1 — Additional file 1: Supplementary Figure S1. Transcriptomic analysis on the NMBA-induced carcinogenesis in ESCC rat model. (a) Enrichment of Gene. Ontology (GO). (b) Enrichment of signaling pathway. The Top 30 enriched terms are shown. (c). Heatmap of chemokines and receptors in carcinogenesis. Microarray data was available on Gene Expression Omnibus (GEO) database under the accession number GSE90464. Supplementary Figure S2. Gating strategy in flow cytometry analysis for tumors. The antibodies used for flow cytometry has been list in Supplementary Table S4. Supplementary Figure S3. Escalating expression of CCL2 in the NMBA-induced ESCC carcinogenesis cell model. (a) Establishment of malignant transformation cell model with NMBA-treatment (10 μM, 25 weeks) in Het-1A cells. (b) Genes expression with the pattern of Continuous increasing over transformation. (c) Enriched pathways for the over-expressed genes. (d) Heatmap shows increased expression of CCL2 and correlated genes. Supplementary Figure S4. ESCC mouse model. (a) CCL2 expression and TAMs accumulation was continuously increase in mouse forestomach during carcinogenesis. (b) Heatmap of Gene expressions by RNA-sequencing with tumors harvested from the CCR2−/− mouse model. Supplementary Figure S5. The effect of CCL2 on pro-death signaling, proliferation and ESCC markers. (a) Heatmap showing relative expression of ESCC markers in normal and ESCC tissues of animal models. (b) The expression of EGFR, BRCA1, CCND1, Myc, Met, TP63, and CD44 were determined with q-PCR in CCL2−/− mouse model. (c) TE-1 cells were treated with CCL2 (0, 10, 50, 500 ng/ml) for 24 h and gene expression was determined by q-PCR. Supplementary Figure S6. Expression of PD-L1 and PD-L2 in polarized macrophages. (a) Differential expression of PD-L1 and PD-L2 in HLA-DR + CD209- M1 macrophages and HLA-DR-CD209+ M2 macrophages. (b) Polarization was not induced by culture with conditioned medium neither by co-culture with cells. THP-1 cells were treate [file 12943_2020_1165_MOESM1_ESM.pdf]

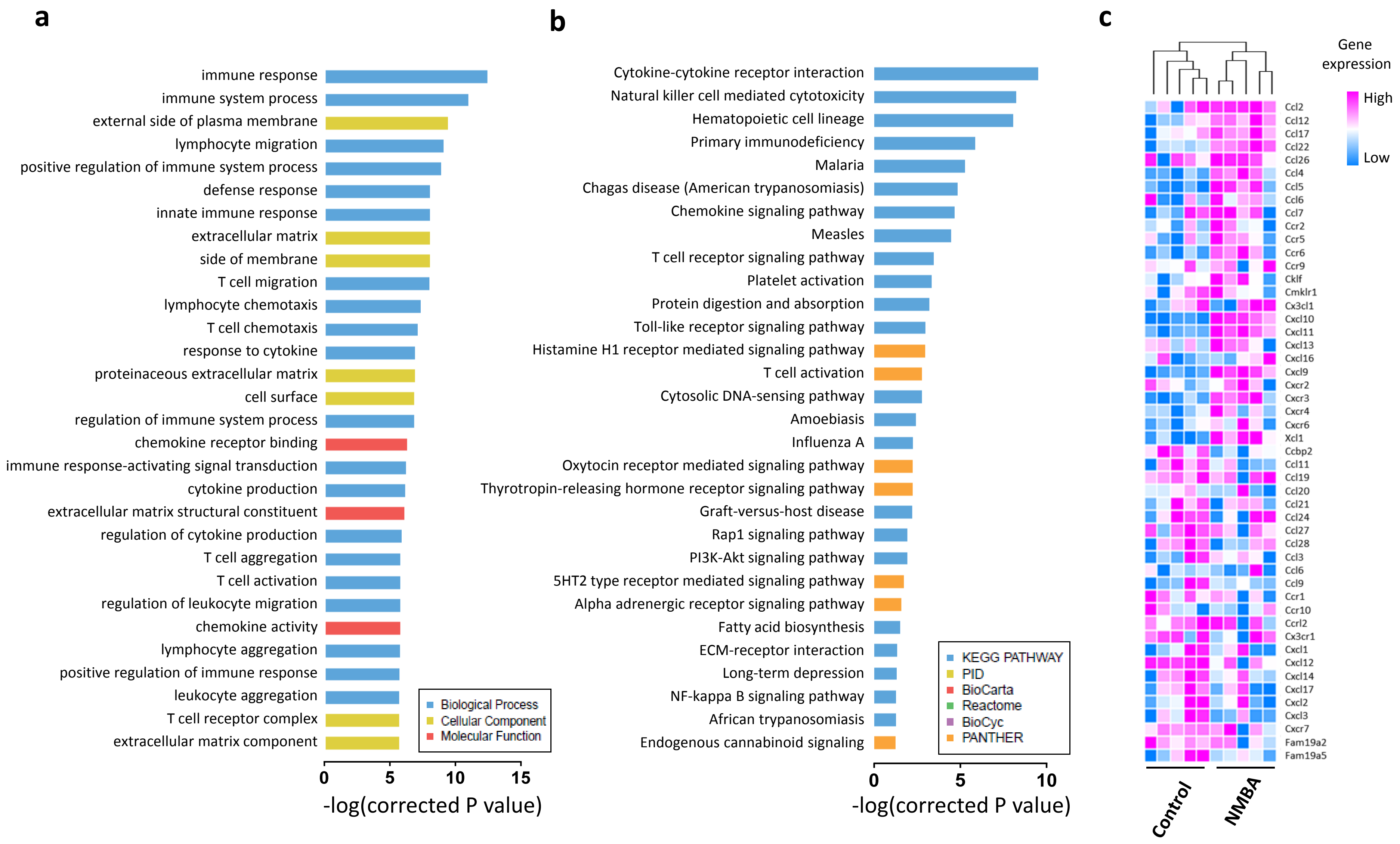

Supplementary figure S1. Transcriptomic analysis on the NMBA-induced carcinogenesis in ESCC rat model. (a) Enrichment of Gene Ontology (GO). (b) Enrichment of signaling pathway. The Top 30 enriched terms are shown. (c) Heatmap of chemokines and receptors in carcinogenesis. Microarray data was available on Gene Expression Omnibus (GEO) database under the accession number GSE90464.

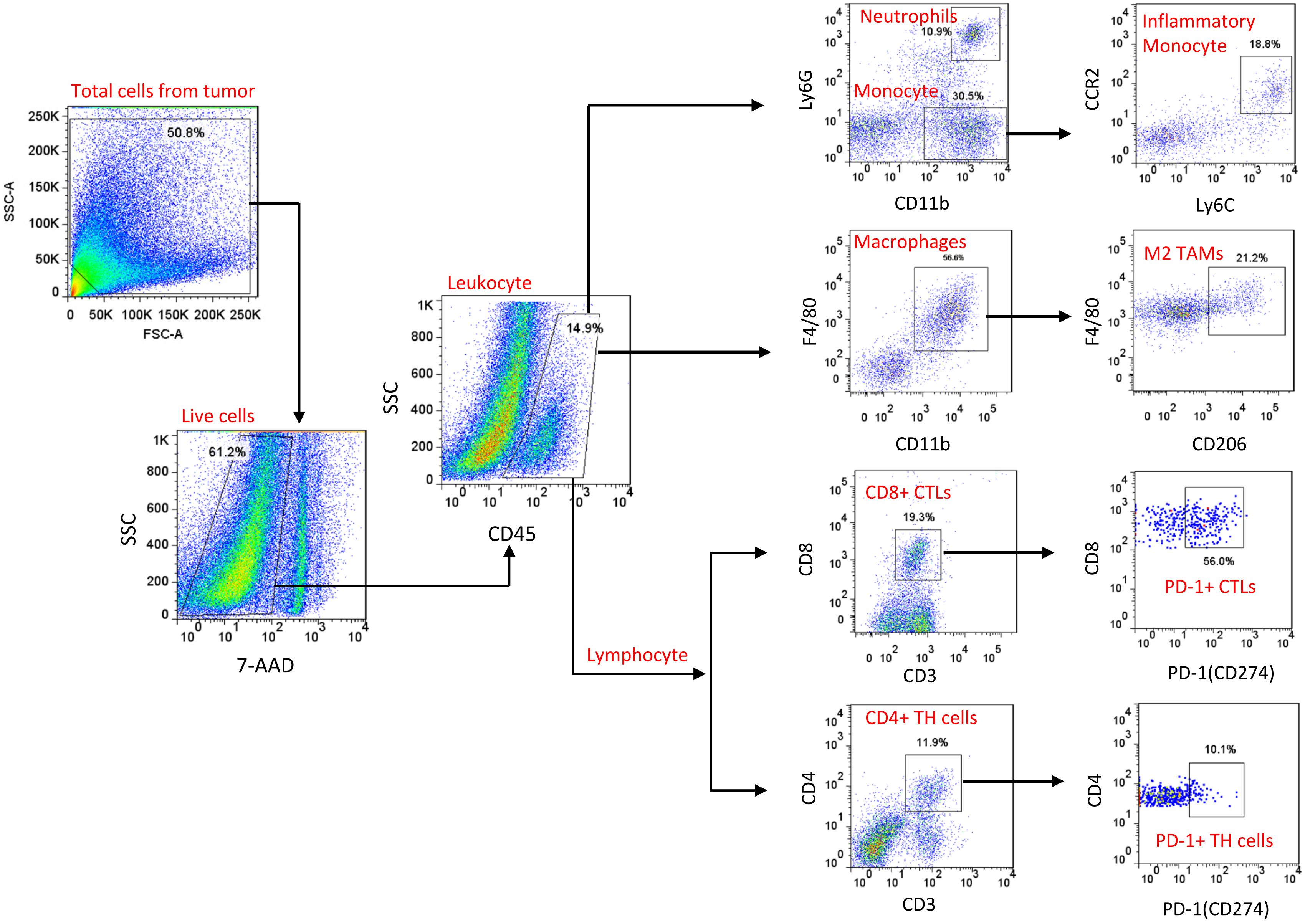

Supplementary figure S2. Gating strategy in flow cytometry analysis for tumors. The antibodies used for flow cytometry has been list in Supplementary table S4.

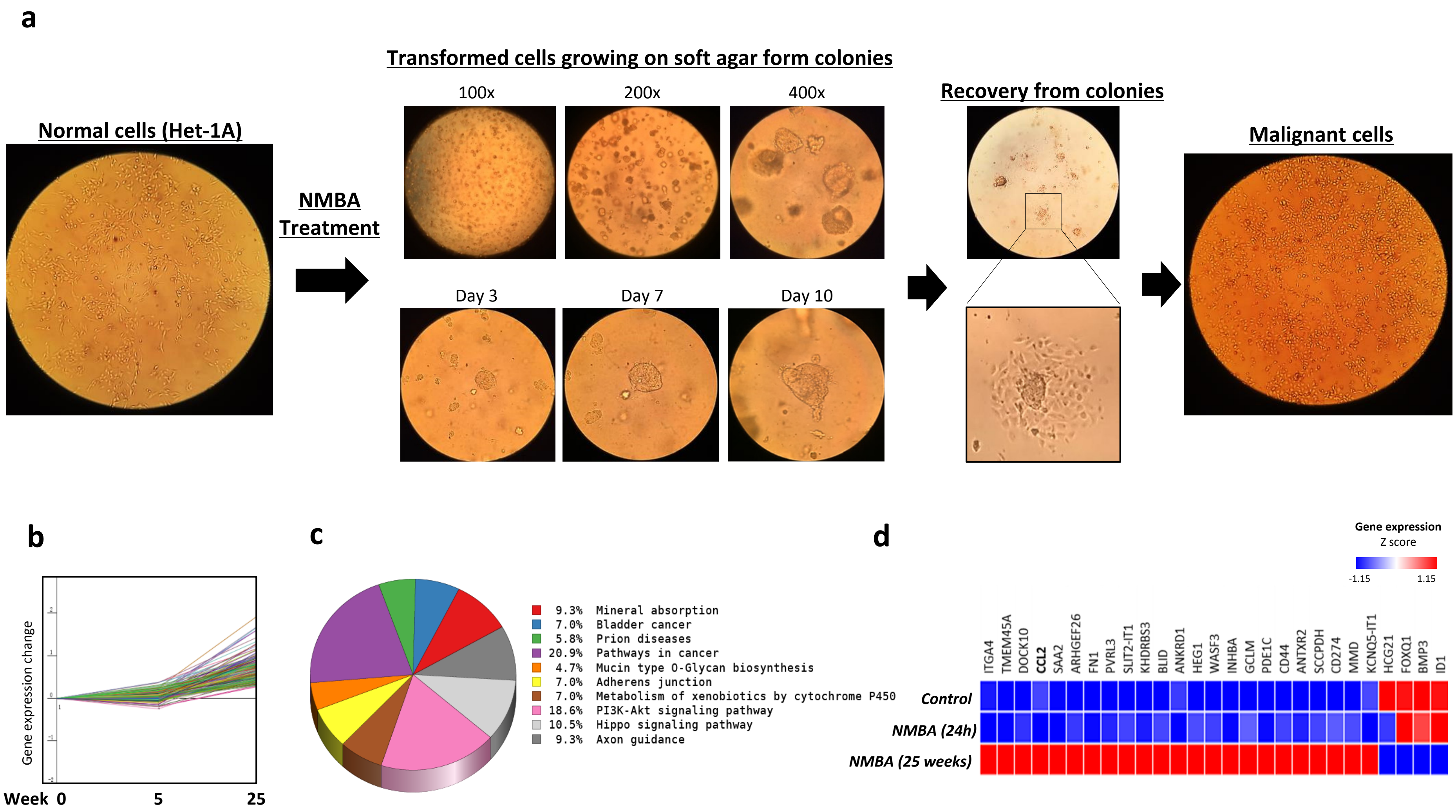

Supplementary figure S3. Escalating expression of CCL2 in the NMBA-induced ESCC carcinogenesis cell model. (a) Establishment of malignant transformation cell model with NMBA-treatment (10μM, 25 weeks) in Het-1A cells. (b) Genes expression with the pattern of Continuous increasing over transformation. (c) Enriched pathways for the over-expressed genes. (d) Heatmap shows increased expression of CCL2 and correlated genes.

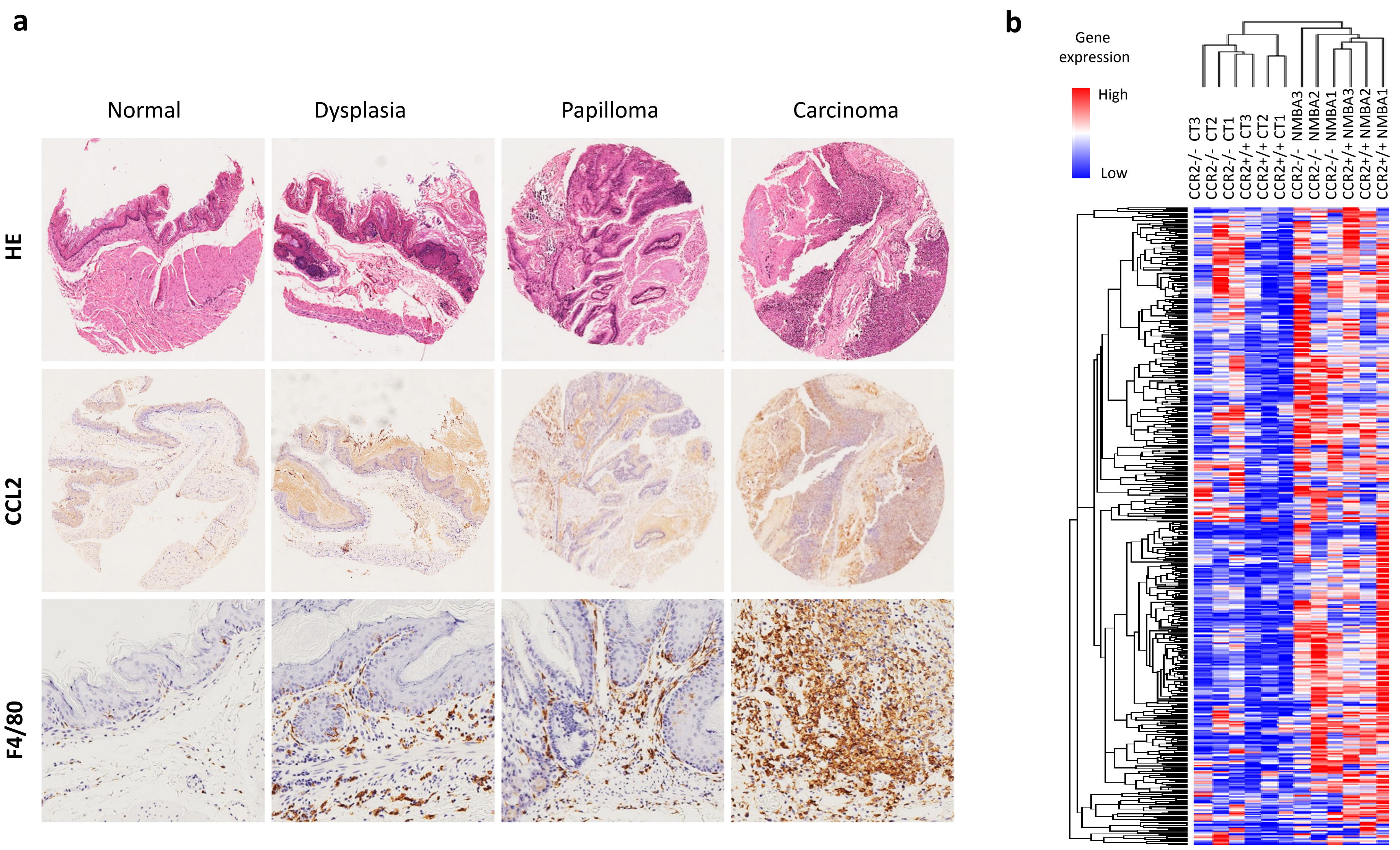

Supplementary figure S4. ESCC mouse model. (a) CCL2 expression and TAMs accumulation was continuously increase in mouse forestomach during carcinogenesis. (b) Heatmap of Gene expressions by RNA-sequencing with tumors harvested from the CCR2-/- mouse model.

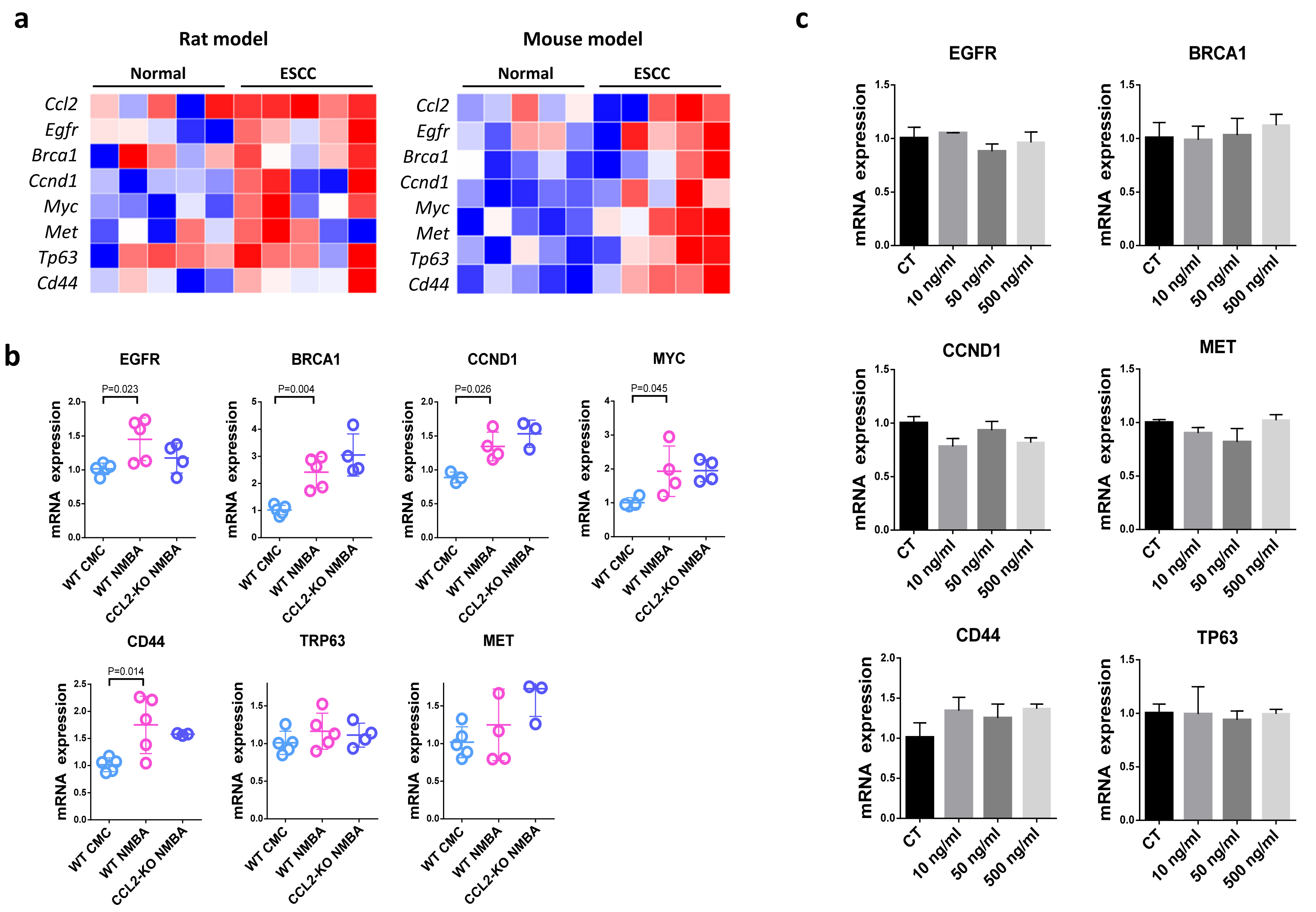

Supplementary figure S5. The effect of CCL2 on pro-death signaling, proliferation and ESCC markers. (a) Heatmap showing relative expression of ESCC markers in normal and ESCC tissues of animal models. (b) The expression of EGFR, BRCA1, CCND1, Myc, Met, TP63, and CD44 were determined with q-PCR in CCL2-/- mouse model. (c) TE-1 cells were treated with CCL2 (0, 10, 50, 500 ng/ml) for 24 hours and gene expression was determined by q-PCR.

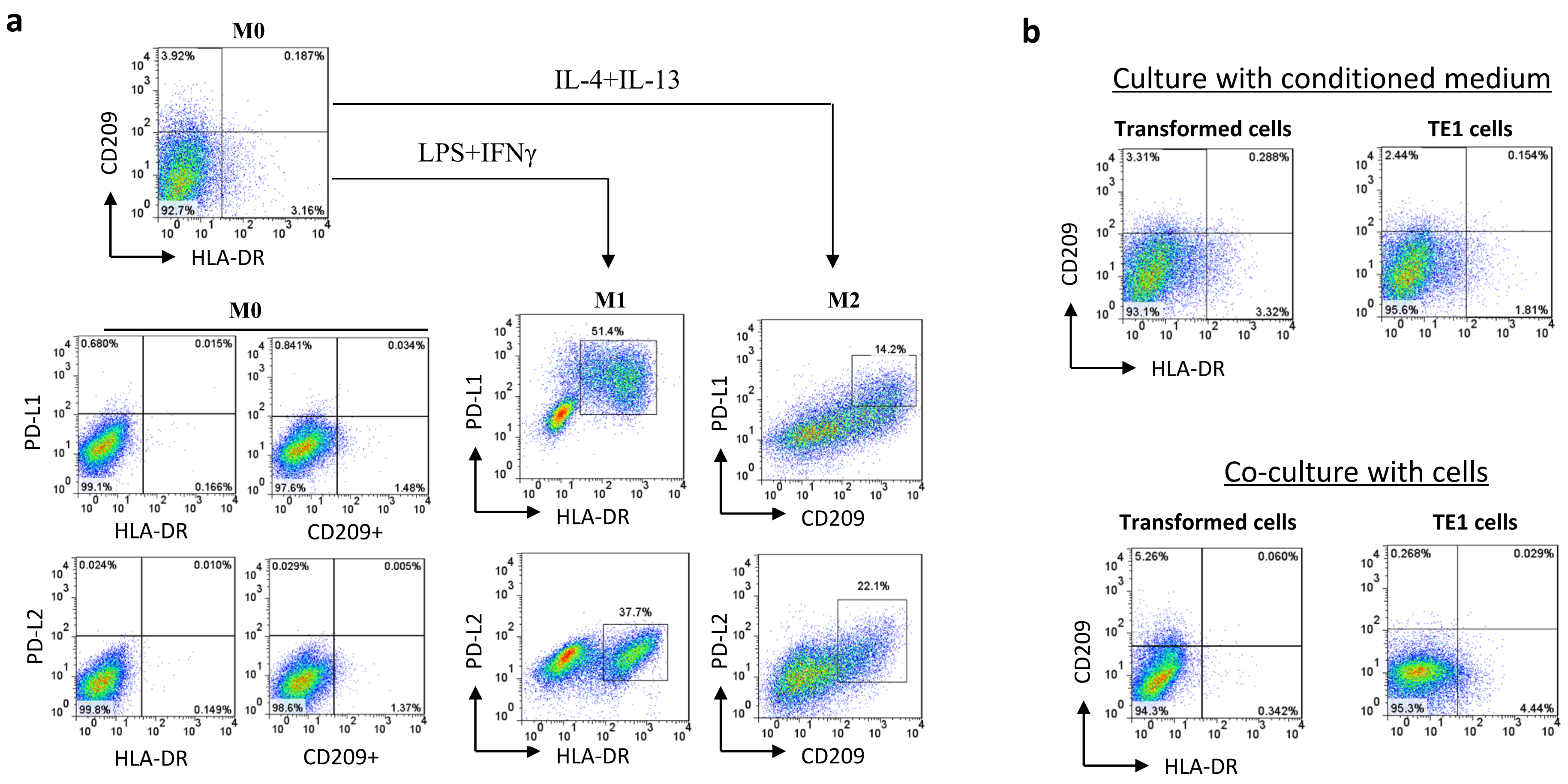

Supplementary figure S6. Expression of PD-L1 and PD-L2 in polarized macrophages. (a) Differential expression of PD-L1 and PD-L2 in HLA-DR$^+$CD209$^-$ M1 macrophages and HLA-DR$^-$CD209$^+$ M2 macrophages. (b) Polarization was not induced by culture with conditioned medium neither by co-culture with cells. THP-1 cells were treated with PMA to induce M0 macrophages as described in Supplementary materials and methods, then incubated with conditioned medium or co-cultured with indicated cells for 72h. Cells were harvested for analysis with flow cytometry.
