# Supplementary material for: CCL2-CCR2 axis recruits tumor associated macrophages to induce immune evasion through PD-1 signaling in esophageal carcinogenesis
Source: Mol Cancer. 2020 Feb 27;19:41. doi: 10.1186/s12943-020-01165-x (PMC7045401; doi:10.1186/s12943-020-01165-x)
Supplement: Supplementary file 2 — Additional file 2. Supplementary materials. Authentication of cell lines. [file 12943_2020_1165_MOESM2_ESM.pdf]

CERTIFICATE OF ANALYSIS

**ATCC® Number:**	**TIB-202™**
**Lot Number:**	**63176297**
**Name:**	**THP-1**
**Description:**	**Acute Monocytic Leukemia**
**Species:**	**Human (*Homo sapiens*)**
**Volume/Ampule:**	**Approximately 1 mL**
**Date Frozen:**	**09JUL2015**
**Recovery:**	**A T-75 setup at a seeding density of 3.0 x 10^5 viable cells/mL is ready to subculture in 3 days.**
**Product Format:**	**Cells cryopreserved in the appropriate cryopreservation medium**
**Expiration Date:**	**Not applicable**
**Storage Conditions:**	**Vapor phase of liquid nitrogen**

Test / Method	Specification	Result
**Ampule passage number**	Report results	Unknown
**Population doubling level (PDL)**	Report results	Not applicable
**Total cells/ampule**<br>(Cell count using Trypan Blue stain method)	Report results	7.2 x 10^6 total cells/ampule
**Post-freeze viability**<br>(Cell count using Trypan Blue stain method)	≥ 50.0%	88.3%
**Growth properties**<br>(Visual observation method)	Suspension	Suspension
**Morphology**<br>(Visual observation method)	Lymphoblast-like*	Lymphoblast-like
**Test for mycoplasma contamination**<br>Hoechst DNA stain (indirect) method<br>Agar culture (direct) method<br>PCR-based assay	 None detected<br>None detected<br>None detected	 None detected<br>None detected<br>None detected
**Species determination: COI assay (interspecies)**	Human	Human

ATCC
10801 University Boulevard
Manassas, VA 20110-2209 USA
www.atcc.org

800-638-6597 or 703-365-2700
Fax: 703-365-2750
E-mail: tech@atcc.org
or contact your local distributor

CERTIFICATE OF ANALYSIS

ATCC® Number: **TIB-202™**

Lot Number: **63176297**

**Species determination: STR analysis (intraspecies)**	**Human (Unique DNA Profile)**<br>TH01: 8, 9.3<br>D5S818: 11, 12<br>D13S317: 13<br>D7S820: 10<br>D16S539: 11, 12<br>CSF1PO: 11, 13<br>Amelogenin: X, Y<br>vWA: 16<br>TPOX: 8, 11	**Human (Unique DNA Profile)**<br>TH01: 8, 9.3<br>D5S818: 11, 12<br>D13S317: 13<br>D7S820: 10<br>D16S539: 11, 12<br>CSF1PO: 11, 13<br>Amelogenin: X, Y<br>vWA: 16<br>TPOX: 8, 11
**Sterility test (BacT/ALERT 3D)**<br>iAST bottle (aerobic) at 32°C<br>iNST bottle (anaerobic) at 32°C	 No growth<br>No growth	 No growth<br>No growth
**Human pathogenic virus testing**<br>(PCR-based assay for HIV, HepB, HPV, EBV, and CMV)	Report results	HIV – None detected<br>HepB – None detected<br>HPV – None detected<br>EBV – None detected<br>CMV – None detected

* Lymphoblast-like: Rounded-like cells in clumps or as single cells in suspension.

Quality Assurance Specialist; Quality Assurance

ATCC hereby represents and warrants that the material provided under this certificate is pure and has been subjected to the tests and procedures specified and that the results described, along with any other data provided in this certificate, are true and correct to the best of the company's knowledge and belief. This certificate does not extend to the growth and/or passage of any living organism or cell line beyond what is supplied within the container received from ATCC.

This product is intended to be used for laboratory research use only. It is not intended for use in humans, animals, or for diagnostics. Appropriate Biosafety Level (BSL) practices should always be used with this material. Refer to the Product Information Sheet for instructions on the correct use of this product.

ATCC products may not be resold, modified for resale, used to provide commercial services, or to manufacture commercial products without prior written agreement from ATCC.

© 2014 American Type Culture Collection. The ATCC trademark and trade name are owned by the American Type Culture Collection.

ATCC
10801 University Boulevard
Manassas, VA 20110-2209 USA
www.atcc.org

800-638-6597 or 703-365-2700
Fax: 703-365-2750
E-mail: tech@atcc.org
or contact your local distributor

CERTIFICATE OF ANALYSIS

ATCC® Number: **CRL-2692™**

Lot Number: **61946858**

Name: **Het-1a**

Description: **Esophageal Epithelium**

Species: **Human (*Homo sapiens*)**

Volume/Ampoule: **Approximately 1 mL**

Date Frozen: **2/26/14**

Recovery: **A T-25 setup at a dilution of 1:10 reaches approximately 70% confluence in 1 day.**
A T-75 setup at a dilution of 1:15 reaches approximately 50-60% confluence in 2 days.

Product Format: **Cells cryopreserved in the appropriate cryopreservation medium**

Expiration Date: **Not applicable**

Storage Conditions: **Vapor phase of liquid nitrogen**

Test / Method	Specification	Result
**Ampule passage number**	Report results	40
**Population doubling level (PDL)**	Report results	N/A
**Total cells/ampoule**<br>(Cell count using Trypan Blue stain method)	Report results	3.4 x 10^{6} total cells/ampoule
**Post-freeze viability**<br>(Cell count using Trypan Blue stain method)	≥ 50.0%	90.3%
**Growth properties**<br>(Visual observation method)	Adherent	Adherent
**Morphology**<br>(Visual observation method)	Epithelial-like*	Epithelial-like
**Test for mycoplasma contamination**<br>Hoechst DNA stain (indirect) method<br>Agar culture (direct) method	 None detected<br>None detected	 None detected<br>None detected
**Species determination: COI assay (interspecies)**	Human	Human

ATCC (American Type Culture Collection)
P.O. Box 1549
Manassas, VA 20108 USA
www.atcc.org

800-638-6597 or 703-365-2700
Fax: 703-365-2750
E-mail: tech@atcc.org
or contact your local distributor

CONFIDENTIAL AND PROPRIETARY
This document contains proprietary information which may not be reproduced, transcribed, or conveyed in any way or for any purpose without the prior written consent of ATCC.
Template Doc ID: 31194 Template Revision: 3 Template Effective Date: 01/31/2013

CERTIFICATE OF ANALYSIS

ATCC® Number: **CRL-2692™**

Lot Number: **61946858**

**Species determination: STR analysis (intraspecies)**	**Human (Unique DNA Profile)**<br>TH01: 7<br>D5S818: 11, 12<br>D13S317: 11<br>D7S820: 9<br>D16S539: 9, 11<br>CSF1PO: 10, 12<br>Amelogenin: X, Y<br>vWA: 16<br>TPOX: 11	**Human (Unique DNA Profile)**<br>TH01: 7<br>D5S818: 11, 12<br>D13S317: 11<br>D7S820: 9<br>D16S539: 9, 11<br>CSF1PO: 10, 12<br>Amelogenin: X, Y<br>vWA: 16<br>TPOX: 11
**Sterility test (BacT/ALERT 3D)**<br>iAST bottle (aerobic) at 32°C<br>iNST bottle (anaerobic) at 32°C	 No growth<br>No growth	 No growth<br>No growth
**Human pathogenic virus testing**<br>(PCR-based assay for HIV, HepB, HPV, EBV, and CMV)	Report results	HIV – not detected<br>HepB – not detected<br>HPV – not detected<br>EBV – not detected<br>CMV – not detected

* Epithelial-like: Any adherent cells of a polygonal shape with clear, sharp boundaries between them.

Quality Assurance Specialist; Quality Assurance

ATCC hereby represents and warrants that the material provided under this certificate is pure and has been subjected to the tests and procedures specified and that the results described, along with any other data provided in this certificate, are true and correct to the best of the company's knowledge and belief. This certificate does not extend to the growth and/or passage of any living organism or cell line beyond what is supplied within the container received from ATCC.

This product is intended to be used for laboratory research use only. It is not intended for use in humans, animals, or for diagnostics. Appropriate Biosafety Level (BSL) practices should always be used with this material. Refer to the Product Information Sheet for instructions on the correct use of this product.

ATCC products may not be resold, modified for resale, used to provide commercial services, or to manufacture commercial products without prior written agreement from ATCC.

The ATCC trademark and trade name and any and all ATCC catalog numbers are trademarks of the American Type Culture Collection.

© 2010 ATCC. All rights reserved.

ATCC (American Type Culture Collection)
P.O. Box 1549
Manassas, VA 20108 USA
www.atcc.org

800-638-6597 or 703-365-2700
Fax: 703-365-2750
E-mail: tech@atcc.org
or contact your local distributor

CONFIDENTIAL AND PROPRIETARY
This document contains proprietary information which may not be reproduced, transcribed, or conveyed in any way or for any purpose without the prior written consent of ATCC.
Template Doc ID: 31194 Template Revision: 3 Template Effective Date: 01/31/2013

The Cell Bank of Type Culture Collection of Chinese Academy of Sciences

STR test report

1. Sample

Cell line: TE-1

Delivery date: 2012/11/13

Report date: 2012/12/3

2. Method

Use Purelink Genomic DNA kits to extract cell DNA and send the DNA to detector.

Detector: Material evidence appraisal center of the ministry of public security

KIT: AmpFISTR® Identifiler® PCR Amplification Kit

Aspirant an appropriate amount of DNA and use the above kit for amplification, then detect the STR loci and sex gene Amelogenin on ABI 3100 genetic analyzer.

3. Results and conclusions

D5S818	11
D13S317	10
D7S820	10,11
D16S539	12
vWA	17,18
TH01	7
Amelogenin	X
TP0X	11
CSF1P0	10,12

The DNA typing of this strain was found in the DSMZ cell bank. Cells matching its cell typing were named TE-1.

No cross-contamination.

Cell bank contact information:

Telephone: 021-54920404, 54920405

Fax: 021-54920406

Contact: Chen songhua, xu huijun

E-mail: shchen@sibs.ac.cn

Address: cell bank, Chinese academy of sciences, no. 320 yueyang road, xuhui district, Shanghai 200031, China

Cell Line Authentication Report

GENEWIZ,Inc. Beijing
30 Science Park Road
Zhong-Guan-Cun Life Science Park
Changping District, 102206
Beijing, China
Tel: 400-8100-669
Fax: 010-59458058
Email: Genomics.China@genewiz.com.cn

www.genewiz.com.cn

GENEWIZ,Inc. Beijing
30 Life Science Park Road
Changping District, 102206
Beijing, China
Tel: 400-8100-669
Fax: 010-59458058
www.genewiz.com.cn

Cell Line Authentication Report

Customer: Liu Yan

Institution: Beijing Hesheng Gene Technology Co Ltd

Quotation Number: GWBJDG1509011

Completion Date：10/14/2015

1. **Sample ID: Het-1A**
2. **Original Material: gDNA**
3. **Methods:**

1).Genomic DNA was extracted from the cell pellets provided by the customer.

2).Samples, together with positive and negative control were amplified using GenePrint 10 System(Promega).

3). Amplified products were processed using the ABI3730xl Genetic Analyzer.

4).Data were analyzed using GeneMapper4.0 software and then compared with the ATCC, DSMZ or JCRB databases for reference matching.

4. **Results:**

1) **10 Loci STR Profile：**

Genetic Site	ATCC		Customer sample	
(Locus)	Het-1A		Het-1A	
Amelogenin	X	Y	X	Y
CSF1PO	10	12	10	12
D13S317	11		11	
D16S539	9	11	9	11
D5S818	11	12	11	12
D7S820	9		9	
THO1	7		7	
TPOX	11		11	
vWA	16		16	
D21S11			28	31
Percent match between the sample and the database profile：100%				

GENEWIZ,Inc. Beijing
30 Life Science Park Road
Changping District, 102206
Beijing, China
Tel: 400-8100-669
Fax: 010-59458058
www.genewiz.com.cn

Summary:

1) Your cell line is considered to be “identical” tothe reference cell line in the ATCC STR database, as the STR profile yields a 100% match.

Notes：

1. P=100% x (2xM)/N; M=13, N=26 P=100% x (2x13)/26=100%

M: number of the matching peaks;   N: number of all peaks

2. Based on the ANSI Standard, cell lines with ≥80% match are considered to be related; i.e.,derived from a common ancestry. Cell lines with between a 55% to 80% match require futher profiling for authentication of relatedness.

3. The short tandem repeat (STR) profile generated by GENEWIZ Inc. is indicative only of the sample sent to GENEWIZ Inc. at the time it was sent. This data and analysis are for research use only.

2) Electrophoretogram

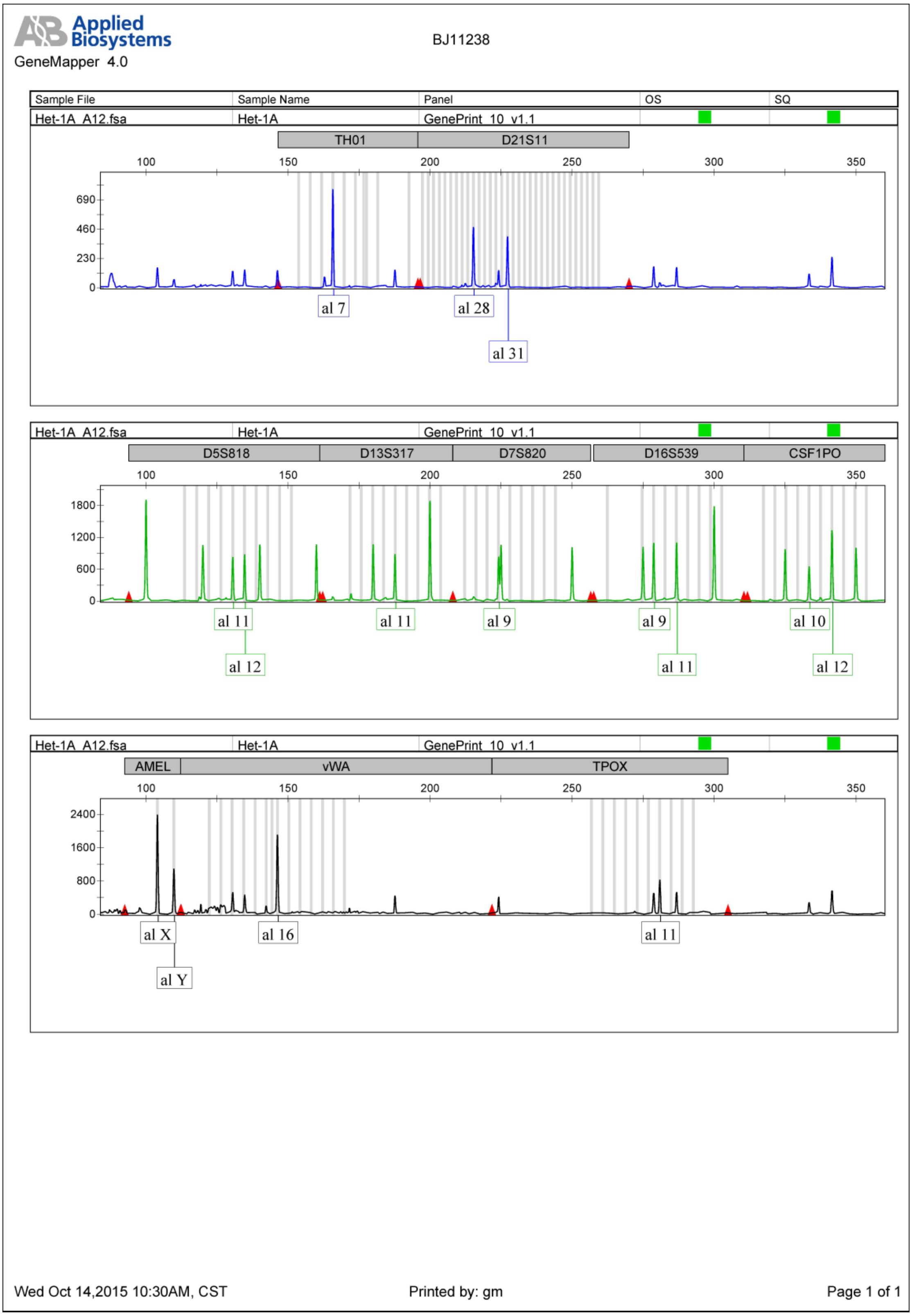

Note：Raw data in appendix
