# Supplementary material for: CCL2-CCR2 axis recruits tumor associated macrophages to induce immune evasion through PD-1 signaling in esophageal carcinogenesis
Source: Mol Cancer. 2020 Feb 27;19:41. doi: 10.1186/s12943-020-01165-x (PMC7045401; doi:10.1186/s12943-020-01165-x)
Supplement: Supplementary file 3 — Additional file 3. Supplementary methods. [file 12943_2020_1165_MOESM3_ESM.pdf]

Supplementary methods

Flow cytometry

Tumors harvested from mice were minced and incubated in Hanks balanced salt solution (HBSS; Sigma-Aldrich) containing 0.2% collagenase type 2 (Worthington) for 60 minutes at 37°C with constant shaking. After inactivating collagenase activity with 10% fetal bovine serum (FBS) containing Dulbecco modified eagle medium (DMEM), the cell suspension was filtered through a 40μm nylon mesh (BD Biosciences), followed by centrifugation at 350 g for 5 minutes. Single cell suspensions were assessed for viability and then incubated with Fc receptor blocking solution, followed by fluorophore-conjugated antibodies. Dead cells were excluded with 7-AAD staining. Flow cytometry was performed on a FACSCalibur (BD Biosciences) and analyzed using FlowJo software. The gating strategies were summarized in Supplementary figure S2.

Quantitative PCR

Total RNA was isolated from rat esophagus and mouse forestomach using the TRIzol reagent, and reverse transcribed using the High Capacity cDNA Archive kit (Thermo, Rockford, IL, USA) following the manufacturers' instructions. Quantitative real-time PCR was performed on a QuantStudio5 PCR System using SYBR green as the detection fluorophore (Thermo, Rockford, IL, USA). Expression of target genes was normalized against that of the housekeeping gene beta-actin. The primer sequences are provided in Supplementary table S3.

Cytokines determination

The protein level of human CCL2 in cell culture medium was determined with Cytometric Bead Array (CBA) CCL2 Flex set (BD). Cytokines in blood or tissue homogenate samples were analyzed with Bead-Based Multiplex Assays using the Luminex technology according to the manufacturer's instructions (Merck Millipore, Darmstadt, Germany).

Immunohistochemistry

Five-micrometer tumor sections were cut from paraffin-embedded tissue and stained with Hematoxylin and Eosin (HE) for histopathological examination. A tumor tissue microarray (TMA) was constructed from 2mm punch biopsies of well-demarcated tumors from embedded tissue blocks. TMA slides were dewaxed in xylene followed by rehydration and antigen retrieval using sodium citrate (10mM). Endogenous peroxidase was quenched by incubation in 3% hydrogen peroxide in PBS. After blocking with 5% normal goat serum, the sections were incubated with primary antibody overnight at 4°C. Negative controls were processed in the absence of the primary antibody. Then HRP-conjugated secondary antibody (Cell signaling) and diaminobenzidine (DAB) were applied to visualize the expression and localization of antigens. Slides were counterstained with Gill's Hematoxylin and photographed with an Aperio VERSA scanning system (Leica Biosystems, Wetzlar, Germany). The IHC staining score was estimated for each sample with intensity value (negative, 0; +, 1; ++, 2; and +++, 3) multiply positive rate value (negative,0; 1-25%, 1; 26-50%, 2; 51-75%, 3; and 76-100%, 4).

Macrophage polarization in vitro

THP-1 monocytes were differentiated into macrophages (M0) by the treatment with Phorbol 12-myristate 13-acetate (PMA, 25ng/ml) for 48 hours. After resting for 24 hours in fresh medium, macrophages were treated with Interferon-gamma (INF-γ, 25ng/ml) and Lipopolysaccharide (LPS, 100ng/ml) for classical macrophage activation (M1), or Interleukin-4 (IL-4, 20ng/ml) and Interleukin-13 (IL-13, 25ng/ml) to obtain M2 polarized macrophages. Cells were harvested for validation with macrophage surface markers CD11b, HLA-DR and CD209 by flow cytometry analysis.

Chemotaxis assay

Chemotaxis of THP-1 cells was determined with Cell Migration Assay Kit that contains polyethylene terephthalate membrane inserts (5μm pore size) in a 24-well plate (Cell Biolabs). Conditioned culture mediums harvested from the transformed Het-1A cells and TE-1 cells were added into the lower well of the plate. THP-1 cell suspension containing 3.0×10^5 cells/ml in serum free media was added in the upper chamber. After incubation for 24h, migratory cells are collected from the lower well and dissociated from the membrane. Subsequently, the cells were quantitatively determined with the patented CyQuant GR Dye (Invitrogen) in a BioTek Synergy 4 plate reader (BioTek).

RNA sequencing

The total RNA (3 μg) was isolated from mouse forestomach tumors with TruSeq RNA Sample Preparation Kit (Illumina, USA). The poly-A containing mRNA molecules were purified from using poly-T oligo-attached magnetic beads (NEB,

USA). The cleaved RNA fragments were reversely transcribed into first strand cDNA using random hexamers, following by second strand cDNA synthesis using DNA Polymerase I and RNase H. Subsequently, the cDNA fragments were purified, end blunted, 'A' tailed, and adaptor ligated. The library was constructed by DNA amplification with PCR and qualified by Agilent 2100 bioanalyzer (Agilent). After quantification using Qubit 2.0 flurometer (Invitrogen, CA, USA) with KAPA Library Quantification Kits (KAPA Biosystem), the generated libraries were sequenced on the HiSeq X platform (Illumina).

Data analysis workflow for RNA sequencing

The sequencing reads were mapped onto the reference gene set with Bowtie software. Reference gene set and annotations (Mus musculus) was obtained from ENSEMBL (https://www.ensembl.org). The mapping result was processed with a Perl script program to generate Gene expression profiles. According to credibility interval approaches reported for the analysis of SAGE data, the edgeR program was used to identify differentially expressed mRNAs [1, 2]. Genes with a P value < 0.05 and expression ratio $>= 2$ (up-regulation) or expression ratio $<= 0.5$ (down-regulation) were recognized as significantly different. Gene Ontology (GO) Enrichments were analyzed in a command-line program KOBAS 2.0. For pathway enrichment analysis, genes were compared with Kyoto Encyclopedia of Genes and Genomes database (KEGG), Reactome Pathway Database, BioCyc Pathway Collection, and PANTHER Pathways Dataset with BLASTX [3]. Gene clustering was performed with Morpheus (https://software.broadinstitute.org/morpheus).

References:

1. Robinson MD, McCarthy DJ, Smyth GK. edgeR: a Bioconductor package for differential expression analysis of digital gene expression data. ***Bioinformatics***. 2010, 26(1): 139-140.
2. Robinson MD, Oshlack A. A scaling normalization method for differential expression analysis of RNA-seq data. ***Genome Biol***. 2010, 11(3): R25.
3. Altschul SF, Madden TL, Schaffer AA, Zhang J, Zhang Z, Miller W, Lipman DJ. Gapped BLAST and PSI-BLAST: a new generation of protein database search programs. ***Nucleic Acids Res***. 1997, 25(17): 3389-3402.
