# Supplementary material for: CCL2-CCR2 axis recruits tumor associated macrophages to induce immune evasion through PD-1 signaling in esophageal carcinogenesis
Source: Mol Cancer. 2020 Feb 27;19:41. doi: 10.1186/s12943-020-01165-x (PMC7045401; doi:10.1186/s12943-020-01165-x)
Supplement: Supplementary file 4 — Additional file 4: Supplementary Table S1. Samples list in the ESCC cohort (100 cases). Supplementary Table S2. ESCC samples list from TCGA database. Supplementary Table S3. Mouse (m) and Human (h) Primers for PCR. Supplementary materials S4. Reagents and antibodies. Supplementary table S5. Uni- and multi-variate COX proportional hazard model analysis for overall survival of ESCC patients in cohort II. Supplementary table S6. KEGG pathway enrichment by GSEA analysis. [file 12943_2020_1165_MOESM4_ESM.pdf]

Supplementary table S1. Samples list in the ESCC cohort (100 cases)

Number	Sample ID	Cancer Type	Sex	Tumor Size (cm)	Overall Survival (Months)	Overall Survival Status	CCL2-IHC Score	CD68 positive cell
1	D08A0181	ESCC	Male	5*2.5*1.5	58	DECEASED	8	150
2	D08A0184	ESCC	Male	4.1*3*1.4	26	DECEASED	2	400
3	D08A0185	ESCC	Male	4.5*3.5*1	13	DECEASED	8	50
4	D08A0187	ESCC	Male	6.5*2.3*2.5	107	LIVING	3	30
5	D08A0188	ESCC	Male	3*2*1.5	15	DECEASED	2	50
6	D08A0189	ESCC	Male	NA	106	LIVING	6	100
7	D08A0194	ESCC	Female	3.5*3.5*1	6	DECEASED	12	40
8	D08A0196	ESCC	Female	7.5*6*1	60	DECEASED	2	60
9	D08A0260	ESCC	Male	3*1.5*1.2	31	DECEASED	2	100
10	D08A0284	ESCC	Male	4.5*3*2	51	DECEASED	8	300
11	D08A0285	ESCC	Female	3.5*2*2	47	DECEASED	6	80
12	D08A0286	ESCC	Male	3*1.5*0.5	29	DECEASED	3	50
13	D08A0290	ESCC	Male	0.5*1.2	22	DECEASED	3	20
14	D08A0293	ESCC	Female	2.5*1.5*1	35	DECEASED	4	90
15	D08A0294	ESCC	Male	4*2*1.5	10	DECEASED	6	30
16	D08A0333	ESCC	Female	5*5*1	100	LIVING	4	400
17	D08A0334	ESCC	Male	4*2*2	2	DECEASED	8	80
18	D08A0337	ESCC	Male	1*1*0.5,1.2*1*1	23	DECEASED	12	150
19	D08A0340	ESCC	Male	7.5*5*1.5	9	DECEASED	2	100
20	D08A0342	ESCC	Male	6*3*1.5	27	DECEASED	12	300
21	D08A0373	ESCC	Male	10*8*2	6	DECEASED	9	60
22	D08A0376	ESCC	Male	5.5*4.5*1.5	43	DECEASED	6	100
23	D08A0379	ESCC	Male	2*2*1	44	DECEASED	6	60
24	D08A0380	ESCC	Male	3*3*1	8	DECEASED	6	50
25	D08A0381	ESCC	Female	5*3*1	9	DECEASED	4	70
26	D08A0383	ESCC	Male	2.5*1.5*0.5	16	DECEASED	4	60
27	D08A0384	ESCC	Male	5*3.5*1.5	97	LIVING	8	200
28	D08A0385	ESCC	Male	6*6*1.5	26	DECEASED	12	50
29	D08A0388	ESCC	Female	4*3*1	97	LIVING	6	150
30	D08A0391	ESCC	Male	6*4*1.5	33	DECEASED	6	300
31	D08A0393	ESCC	Female	5*4*1	10	DECEASED	9	150
32	D08A0394	ESCC	Male	4*2.5*1.5	6	DECEASED	12	200
33	D08A0395	ESCC	Male	3*1.5*1	8	DECEASED	8	80
34	D08A0397	ESCC	Male	NA	2	DECEASED	12	200
35	D08A0400	ESCC	Male	5*3.5*1	43	DECEASED	8	200
36	D08A0401	ESCC	Male	4*3*1	44	DECEASED	6	300
37	D08A0402	ESCC	Male	3*2.5*1	95	LIVING	4	40
38	D08A0403	ESCC	Male	4.5*3*0.6	23	DECEASED	6	80
39	D08A0404	ESCC	Male	3*3*3	73	LIVING	6	80
40	D08A0405	ESCC	Male	8*4*2	4	DECEASED	8	200
41	D08A0477	ESCC	Male	4*2*1，6*5*4	9	DECEASED	12	300
42	D08A0569	ESCC	Male	7*3.5*2	13	DECEASED	0	10
43	D08A0571	ESCC	Male	5*2*1	15	DECEASED	0	70
44	D08A0572	ESCC	Male	6*4*1	13	DECEASED	9	100
45	D08A0573	ESCC	Female	4.5*3*1.5	2	DECEASED	6	60
46	D08A0577	ESCC	Female	4*3*1.5	88	LIVING	2	100
47	D08A0579	ESCC	Male	5*3.5*1.5	33	DECEASED	9	150
48	D08A0580	ESCC	Male	2.5*2*0.5	12	DECEASED	9	400

49	D08A0581	ESCC	Female	5*4*2	12	DECEASED	4	200
50	D08A0582	ESCC	Male	6*5*1.5 ；	88	LIVING	4	100
51	D08A0583	ESCC	Male	3.8*2*1	4	DECEASED	12	150
52	D08A0584	ESCC	Male	3*2.5*1	5	DECEASED	12	200
53	D08A0588	ESCC	Male	4*2.5*1	30	DECEASED	6	20
54	D08A0589	ESCC	Male	NA	15	DECEASED	4	20
55	D08A0593	ESCC	Male	4*4*1	87	LIVING	4	10
56	D08A0595	ESCC	Female	NA	5	DECEASED	8	100
57	D08A0596	ESCC	Female	6*5*1	22	DECEASED	9	100
58	D08A0648	ESCC	Female	4*2*1	5	DECEASED	8	300
59	D08A0649	ESCC	Male	5*3*1	0	DECEASED	9	200
60	D08A0650	ESCC	Male	7*3*1	5	DECEASED	12	100
61	D08A0652	ESCC	Male	NA	86	LIVING	9	300
62	D08A0659	ESCC	Male	NA	7	DECEASED	8	40
63	D08A0660	ESCC	Female	NA	14	DECEASED	12	100
64	D08A0692	ESCC	Male	4*2*1	15	DECEASED	12	20
65	D08A0694	ESCC	Male	7.5*4*1.5	5	DECEASED	12	100
66	D08A0696	ESCC	Female	3*2*1	16	DECEASED	6	30
67	D08A0697	ESCC	Male	4.5*3*1	84	LIVING	4	150
68	D08A0699	ESCC	Male	6*6*1.5	26	DECEASED	12	200
69	D08A0700	ESCC	Male	7*4*1.5	84	LIVING	3	50
70	D08A0701	ESCC	Female	5.5*3*1.5	84	LIVING	8	300
71	D08A0702	ESCC	Male	4*3*1	1	DECEASED	8	200
72	D08A0705	ESCC	Male	NA	83	LIVING	9	300
73	D08A0718	ESCC	Female	5.5*4*1.5	5	DECEASED	9	20
74	D08A0719	ESCC	Female	2*1*0.8	83	LIVING	9	200
75	D08A0720	ESCC	Male	NA	4	DECEASED	12	100
76	D08A0721	ESCC	Male	7*4*1	15	DECEASED	12	400
77	D08A0722	ESCC	Male	10*3*1	55	DECEASED	6	150
78	D08A0723	ESCC	Male	1.5*1*1	25	DECEASED	4	300
79	D08A0724	ESCC	Female	NA	10	DECEASED	6	90
80	D08A0745	ESCC	Male	NA	27	DECEASED	12	60
81	D08A0746	ESCC	Male	5*3*2	82	LIVING	6	400
82	D08A0748	ESCC	Male	6*4.5*1.5	81	LIVING	9	50
83	D08A0752	ESCC	Female	NA	28	DECEASED	6	100
84	D08A0753	ESCC	Male	5*4*2	10	DECEASED	3	20
85	D08A0803	ESCC	Male	4.5*3.7*0.5	83	LIVING	4	200
86	D08A0804	ESCC	Male	NA	10	DECEASED	6	80
87	D08A1719	ESCC	Female	6*3.5*0.7	10	DECEASED	9	150
88	D08A1720	ESCC	Female	4*2*2	31	DECEASED	9	50
89	D08A1729	ESCC	Male	NA	15	DECEASED	12	100
90	D08A1731	ESCC	Male	5.5*3*1.5	19	DECEASED	4	60
91	D08A1732	ESCC	Male	2*2*1	10	DECEASED	9	200
92	D08A1735	ESCC	Male	NA	24	DECEASED	6	60
93	D08A1740	ESCC	Female	6*3*1.5	9	DECEASED	6	50
94	D08A1742	ESCC	Male	7*4*0.8	8	DECEASED	6	20
95	D08A1743	ESCC	Male	5*4*1.5	27	DECEASED	9	150
96	D08A1746	ESCC	Male	3*3*1	23	DECEASED	6	50
97	D08A1747	ESCC	Female	1.5*1*0.5	4	DECEASED	6	100
98	D08A1750	ESCC	Female	6*3.3*1	10	DECEASED	12	200
99	D08A1751	ESCC	Male	3*2*1	19	DECEASED	12	100
100	D08A1755	ESCC	Male	5.5*3*1.5	8	DECEASED	6	100

Supplementary table S2. ESCC samples list from TCGA database

Number	Sample ID	Cancer Type	Sex	AJCC Stage	Histologic Grade	Overall Survival (Months)	Overall Survival Status	Informed consent verified
1	TCGA-IG-A3I8-01	ESCC	Female	T3	G2	33.25	LIVING	YES
2	TCGA-IG-A3QL-01	ESCC	Male	T2	G2	35.18	LIVING	YES
3	TCGA-IG-A3Y9-01	ESCC	Male	T4	G2	0.85	DECEASED	YES
4	TCGA-IG-A3YA-01	ESCC	Male	T4	G2	20.76	LIVING	YES
5	TCGA-IG-A3YB-01	ESCC	Male	T3	G2	2.63	LIVING	YES
6	TCGA-IG-A3YC-01	ESCC	Male	T3	G2	20.11	LIVING	YES
7	TCGA-IG-A4P3-01	ESCC	Male	T2	G2	18.63	DECEASED	YES
8	TCGA-IG-A50L-01	ESCC	Male	T3	G2	0.53	LIVING	YES
9	TCGA-IG-A51D-01	ESCC	Male	T1	G2	17.02	LIVING	YES
10	TCGA-IG-A5B8-01	ESCC	Male	T3	G1	0.79	DECEASED	YES
11	TCGA-IG-A5S3-01	ESCC	Female	T3	G2	23.39	LIVING	YES
12	TCGA-IG-A625-01	ESCC	Male	T3	G2	12.81	DECEASED	YES
13	TCGA-IG-A6QS-01	ESCC	Male	T2	G1	9.95	DECEASED	YES
14	TCGA-IG-A8O2-01	ESCC	Male	T3	G3	4.66	DECEASED	YES
15	TCGA-IG-A97H-01	ESCC	Male	T3	G3	14.49	LIVING	YES
16	TCGA-IG-A97I-01	ESCC	Male	T2	G2	12.16	LIVING	YES
17	TCGA-JY-A6FD-01	ESCC	Female	T3	G1	67.97	LIVING	YES
18	TCGA-JY-A6FE-01	ESCC	Male	T3	G3	3.68	DECEASED	YES
19	TCGA-JY-A6FG-01	ESCC	Male	T3	G2	41.49	DECEASED	YES
20	TCGA-JY-A93F-01	ESCC	Female	T2	G1	24.01	LIVING	YES
21	TCGA-KH-A6WC-01	ESCC	Male	T1	GX	6.27	LIVING	YES
22	TCGA-L5-A43H-01	ESCC	Male	T3	GX	0.3	DECEASED	YES
23	TCGA-L5-A43J-01	ESCC	Male	T3	G3	4.3	DECEASED	YES
24	TCGA-L5-A4OM-01	ESCC	Female	T1	GX	47.9	DECEASED	YES
25	TCGA-L5-A88S-01	ESCC	Male	T3	GX	15.47	LIVING	YES
26	TCGA-L5-A88W-01	ESCC	Male	T3	GX	25.1	DECEASED	YES
27	TCGA-L5-A88Z-01	ESCC	Female	T1	GX	7.39	LIVING	YES
28	TCGA-L5-A8NK-01	ESCC	Female	T3	G1	13.53	LIVING	YES
29	TCGA-L5-A8NQ-01	ESCC	Male	T2	G3	21.35	DECEASED	YES
30	TCGA-L7-A56G-01	ESCC	Male	NA	G3	10.84	DECEASED	YES
31	TCGA-LN-A49K-01	ESCC	Male	T3	G2	5.91	DECEASED	YES
32	TCGA-LN-A49L-01	ESCC	Male	T2	G2	10.45	DECEASED	YES
33	TCGA-LN-A49M-01	ESCC	Male	T2	G1	12.65	LIVING	YES
34	TCGA-LN-A49N-01	ESCC	Male	T2	G2	12.42	LIVING	YES
35	TCGA-LN-A49O-01	ESCC	Male	T3	G3	13.4	LIVING	YES
36	TCGA-LN-A49P-01	ESCC	Male	T3	G2	12.32	LIVING	YES
37	TCGA-LN-A49R-01	ESCC	Male	T3	G3	13.37	LIVING	YES
38	TCGA-LN-A49S-01	ESCC	Male	T3	G2	13.14	LIVING	YES
39	TCGA-LN-A49U-01	ESCC	Male	T3	G1	15.34	LIVING	YES
40	TCGA-LN-A49W-01	ESCC	Male	T3	G3	13.24	LIVING	YES
41	TCGA-LN-A49X-01	ESCC	Male	T3	G2	12.61	LIVING	YES
42	TCGA-LN-A49Y-01	ESCC	Male	T3	G2	12.45	LIVING	YES
43	TCGA-LN-A4A1-01	ESCC	Male	T3	G3	12.58	LIVING	YES

44	TCGA-LN-A4A2-01	ESCC	Male	T3	G1	12.48	LIVING	YES
45	TCGA-LN-A4A3-01	ESCC	Male	T3	G2	12.75	LIVING	YES
46	TCGA-LN-A4A4-01	ESCC	Male	T3	GX	12.58	LIVING	YES
47	TCGA-LN-A4A5-01	ESCC	Male	T2	G2	22.37	DECEASED	YES
48	TCGA-LN-A4A6-01	ESCC	Male	T2	G2	12.84	LIVING	YES
49	TCGA-LN-A4A8-01	ESCC	Male	T2	G2	15.51	LIVING	YES
50	TCGA-LN-A4A9-01	ESCC	Male	T2	G1	11.53	DECEASED	YES
51	TCGA-LN-A4MQ-01	ESCC	Male	T3	G3	12.32	LIVING	YES
52	TCGA-LN-A4MR-01	ESCC	Male	T2	G2	13.21	LIVING	YES
53	TCGA-LN-A5U5-01	ESCC	Male	T3	G2	4.47	DECEASED	YES
54	TCGA-LN-A5U6-01	ESCC	Male	T2	G2	12.32	LIVING	YES
55	TCGA-LN-A5U7-01	ESCC	Male	T2	G1	25.23	LIVING	YES
56	TCGA-LN-A7HV-01	ESCC	Male	T2	G1	10.51	LIVING	YES
57	TCGA-LN-A7HW-01	ESCC	Male	T2	G2	11.99	LIVING	YES
58	TCGA-LN-A7HX-01	ESCC	Male	T2	G2	12.22	LIVING	YES
59	TCGA-LN-A7HY-01	ESCC	Male	T3	G2	12.02	LIVING	YES
60	TCGA-LN-A7HZ-01	ESCC	Male	T2	G2	13.17	LIVING	YES
61	TCGA-LN-A8HZ-01	ESCC	Male	T2	G3	12.32	LIVING	YES
62	TCGA-LN-A8I0-01	ESCC	Male	T2	G2	13.37	LIVING	YES
63	TCGA-LN-A8I1-01	ESCC	Female	T2	G3	13.17	LIVING	YES
64	TCGA-LN-A9FO-01	ESCC	Male	T2	G2	0.13	LIVING	YES
65	TCGA-LN-A9FP-01	ESCC	Female	T2	G3	12.02	LIVING	YES
66	TCGA-LN-A9FQ-01	ESCC	Male	T3	G3	12.84	LIVING	YES
67	TCGA-LN-A9FR-01	ESCC	Male	T2	G2	12.25	LIVING	YES
68	TCGA-Q9-A6FU-01	ESCC	Female	T3	G2	5.16	DECEASED	YES
69	TCGA-S8-A6BW-01	ESCC	Male	T2	G1	20.37	LIVING	YES
70	TCGA-V5-A7RC-01	ESCC	Male	NA	GX	3.42	DECEASED	YES
71	TCGA-V5-AASV-01	ESCC	Male	T3	G3	15.34	LIVING	YES
72	TCGA-VR-A8EO-01	ESCC	Male	T3	G3	25.79	LIVING	YES
73	TCGA-VR-A8EP-01	ESCC	Male	T3	G3	27.07	LIVING	YES
74	TCGA-VR-A8ER-01	ESCC	Male	T4	GX	12.42	DECEASED	YES
75	TCGA-VR-A8ET-01	ESCC	Male	T2	G2	1.54	DECEASED	YES
76	TCGA-VR-A8EU-01	ESCC	Male	T1	G2	18.3	DECEASED	YES
77	TCGA-VR-A8EW-01	ESCC	Male	T3	G2	8.11	DECEASED	YES
78	TCGA-VR-A8EX-01	ESCC	Male	T1	G2	28.09	DECEASED	YES
79	TCGA-VR-A8EY-01	ESCC	Female	T3	G1	33.67	LIVING	YES
80	TCGA-VR-A8EZ-01	ESCC	Male	T3	G2	18.17	DECEASED	YES
81	TCGA-VR-AA4G-01	ESCC	Female	T2	G2	18.04	LIVING	YES
82	TCGA-VR-AA7B-01	ESCC	Female	T3	G2	11.24	LIVING	YES
83	TCGA-VR-AA7D-01	ESCC	Male	T3	G2	9.17	DECEASED	YES
84	TCGA-VR-AA7I-01	ESCC	Male	T4	G1	15.9	DECEASED	YES
85	TCGA-XP-A8T6-01	ESCC	Male	T2	G2	25.07	DECEASED	YES
86	TCGA-XP-A8T8-01	ESCC	Male	T1	G1	14.36	LIVING	YES
87	TCGA-Z6-A8JD-01	ESCC	Male	T3	G2	3.42	LIVING	YES
88	TCGA-Z6-A8JE-01	ESCC	Male	T3	G3	2.1	LIVING	YES
89	TCGA-Z6-A9VB-01	ESCC	Male	T3	G2	1.31	LIVING	YES
90	TCGA-Z6-AAPN-01	ESCC	Male	T3	G1	2.66	LIVING	YES

Supplementary table S3. Mouse (m) and Human (h) Primers for PCR

Primers	Sequence (5' to 3')
mCCL2-F	GCATCCACGTGTTGGCTCAG
mCCL2-R	TTCTTGGGGTCAGCACAGAC
mCCR2-F	TCTTCCTGCTCACATTACCA
mCCR2-R	GCCAAGTACCTATCAATTGT
mIL-12b-F	TTGAACTGGCGTTGGAAGCACG
mIL-12b-R	CCACCTGTGAGTTCTTCAAAGGC
mIL-13-F	AACGGCAGCATGGTATGGAGTG
mIL-13-R	TGGGTCCTGTAGATGGCATTGC
mIL-10-F	CGGGAAGACAATAACTGCACCC
mIL-10-R	CGGTTAGCAGTATGTTGTCCAGC
mPD1-F	CCCAAGGCAAAAATCGAGGAGAG
mPD1-R	CGAGGGGCTGGGATATCTTG
mPD-L1-F	AGATCACAGCCAGGGCAAAA
mPD-L1-R	AGAAGAGGAGGACCGTGGAC
mPD-L2-F	CTCGTTCCACATACCTCAAGTCC
mPD-L2-R	CTGGAACCTTTAGGATGTGAGTG
mMet-F	GTTCTGCTTGGCAACGAGAGCT
mMet-R	GGAGAATGCACTGTATTGCGTCG
hMET-F	TGCACAGTTGGTCCTGCCATGA
hMET-R	CAGCCATAGGACCGTATTTCGG
hEGFR-F	AACACCCTGGTCTGGAAGTACG
hEGFR-R	TCGTTGGACAGCCTTCAAGACC
mEgfr-F	GGACTGTGTCTCCTGCCAGAAT
mEgfr-R	GGCAGACATTCTGGATGGCACT
mBrca1-F	CGAGGAAATGGCAACTTGCCTAG
mBrca1-R	TCACTCTGCGAGCAGTCTTCAG
hBRCA1-F	CTGAAGACTGCTCAGGGCTATC
hBRCA1-R	AGGGTAGCTGTTAGAAGGCTGG
mTrp63-F	GTATCGGACAGCGCAAAGAACG
mTrp63-R	CTGGTAGGTACAGCAGCTCATC
hTP63-F	CAGGAAGACAGAGTGTGCTGGT
hTP63-R	AATTGGACGGCGGTTCATCCCT
mSox2-F	AACGGCAGCTACAGCATGATGC
mSox2-R	CGAGCTGGTCATGGAGTTGTAC
hSOX2-F	GCTACAGCATGATGCAGGACCA
hSOX2-R	TCTGCGAGCTGGTCATGGAGTT
mMyc-F	TCGCTGCTGTCCTCCGAGTCC
mMyc-R	GGTTTGCCTCTTCTCCACAGAC
hMYC-F	CCTGGTGCTCCATGAGGAGAC
hMYC-R	CAGACTCTGACCTTTTGCCAGG
mCd44-F	CGGAACCACAGCCTCCTTTCAA
mCd44-R	TGCCATCCGTTCTGAAACCACG
hCD44-F	CCAGAAGGAACAGTGGTTTGGC
hCD44-R	ACTGTCCTCTGGGCTTGGTGTT

Supplementary materials S4. Reagents and antibodies

Catlog #	Reagent Name	Clone	Vendor	Application
CC-3170	BEGM Bullet Kit	Not applied	Lonza	Cell culture
CC-5002	Trypsin Neutralizing Solution	Not applied	Lonza	Cell culture
279-MC-010	Recombinant Human CCL2/MCP-1 Protein	Not applied	R&D	Chemotaxis
CBA-102	CytoSelect 24-Well Cell Migration Assay kit (5µm, Fluorometric Format)	Not applied	Cell biolabs	Chemotaxis
P8139	Phorbol 12-myristate 13-acetate	Not applied	Sigma-Aldrich	Macrophage polarization
L6529	Lipopolysaccharides from Escherichia coli O55:B5	Not applied	Sigma-Aldrich	Macrophage polarization
300-02	Recombinant Human IFN-gamma	Not applied	Peprotech	Macrophage polarization
200-04	Recombinant Human IL-4	Not applied	Peprotech	Macrophage polarization
200-13	Recombinant human IL-13	Not applied	Peprotech	Macrophage polarization
LS004176	Collagenase, Type 2	Not applied	Worthington	Cell isolation
553080	FITC Rat Anti-Mouse CD45 Antibody	30-F11	BD Pharmingen	Flow cytometry
560527	PerCP-Cy™5.5 Rat Anti-Mouse CD3 Molecular Complex Antibody	17A2	BD Pharmingen	Flow cytometry
558694	APC Mouse anti-Human CD279 Antibody	MIH4	BD Pharmingen	Flow cytometry
553049	PE Rat Anti-Mouse CD4 Antibody	RM4-5	BD Pharmingen	Flow cytometry
565410	PE Rat Anti-Mouse F4/80 Antibody	T45-2342	BD Pharmingen	Flow cytometry
550416	Purified Mouse Anti-Human MCP-1 Antibody	5D3-F7	BD Pharmingen	Flow cytometry
100708	PE anti-mouse CD8a Antibody	53-6.7	Biolegend	Flow cytometry
101228	PerCP/Cy5.5 anti-mouse/human CD11b Antibody	M1/70	Biolegend	Flow cytometry
103132	PerCP/Cy5.5 anti-mouse CD45 Antibody	30-F11	Biolegend	Flow cytometry
107210	APC anti-mouse CD273 (B7-DC, PD-L2) Antibody	TY25	Biolegend	Flow cytometry
108909	APC anti-mouse CD49b (pan-NK cells) Antibody	DX5	Biolegend	Flow cytometry
115507	PE anti-mouse CD19 Antibody	6D5	Biolegend	Flow cytometry
123110	PE anti-mouse F4/80 Antibody	BM8	Biolegend	Flow cytometry
124312	APC anti-mouse CD274 (B7-H1, PD-L1) Antibody	10F.9G2	Biolegend	Flow cytometry
127614	APC anti-mouse Ly-6G Antibody	1A8	Biolegend	Flow cytometry
128008	PE anti-mouse Ly-6C Antibody	HK1.4	Biolegend	Flow cytometry

135210	APC anti-mouse CD279 (PD-1) Antibody	29F.1A12	Biolegend	Flow cytometry
141708	APC anti-mouse CD206 (MMR) Antibody	C068C2	Biolegend	Flow cytometry
150604	Alexa Fluor 647 anti-mouse CD192 (CCR2) Antibody	SA203G11	Biolegend	Flow cytometry
307627	PerCP anti-human HLA-DR Antibody	L243	Biolegend	Flow cytometry
329708	APC anti-human CD274 (B7-H1, PD-L1) Antibody	29E.2A3	Biolegend	Flow cytometry
330106	PE anti-human CD209 (DC-SIGN) Antibody	9E9A8	Biolegend	Flow cytometry
422302	Human TruStain FcX™ (Fc Receptor Blocking Solution)	Not applied	Biolegend	Flow cytometry
84651S	PD-1 Rabbit monoclonal antibody (Mouse Specific)	D7D5W	CST	IHC
98941S	CD8α Rabbit monoclonal antibody (Mouse Specific)	D4W2Z	CST	IHC
83882S	TIM-3 Rabbit monoclonal antibody (Mouse Specific)	D3M9R	CST	IHC
30325S	F4/80 Rabbit monoclonal antibody	D4C8V	CST	IHC
93668S	Arginase-1 Rabbit monoclonal antibody	D4E3M	CST	IHC
8125S	SignalStain Boost IHC Detection Reagent (HRP, Mouse)	Not applied	CST	IHC
MAB2791-100	Human CCL2/MCP-1 Antibody	23002	R&D	IHC
MAB2040	Human CD68/SR-D1 Antibody	298813	R&D	IHC
MAB55381	Mouse CCR2 Antibody	475301	R&D	IHC
MCA341GA	Mouse anti-rat CD68 Antibody	ED1	Bio-Rad	IHC
ab7202	Anti-MCP1/CCL2 antibody	Polyclonal	Abcam	IHC
RECYTMAG-65K	MILLIPLEX MAP Rat Cytokine/Chemokine Magnetic Bead Panel	Not applied	Millipore	Immunology Multiplex Assay
MCYTOMAG-70K	MILLIPLEX MAP Mouse Cytokine/Chemokine Magnetic Bead Panel	Not applied	Millipore	Immunology Multiplex Assay
740007	LEGENDplex™ Mouse Proinflammatory Chemokine Panel	Not applied	Biolegend	Immunology Multiplex Assay
740502	LEGENDplex™ Human Macrophage/Microglia Panel	Not applied	Biolegend	Immunology Multiplex Assay
740845	LEGENDplex™ Mouse Macrophage/Microglia Panel	Not applied	Biolegend	Immunology Multiplex Assay
AM9932	Nuclease-Free Water	Not applied	Life Technologies	PCR
A25742	PowerUp™ SYBR™ Green Master Mix	Not applied	Life Technologies	PCR
74004	RNeasy Micro Kit	Not applied	Qiagen	PCR

Supplementary table S5. Uni- and multi-variate COX proportional hazard model analysis for overall survival of ESCC patients in cohort II

Variable		Univariate		multivariate	
		HR(95%CI)	P value	HR(95%CI)	P value
Gender			0.865		0.637
	Male	0.958(0.582-1.576)	0.865	1.155(0.635-2.099)	0.637
Age			0.055		0.104
	>65	1.539(0.990-2.392)	0.055	1.549(0.914-2.626)	0.104
Clinical grade[1]			0.954		0.906
	II	0.922(0.547-1.554)	0.760	0.864(0.449-1.663)	0.662
	III	0.966(0.554-1.683)	0.902	0.892(0.431-1.847)	0.758
T stage[2]			0.241		0.936
	T3 or T4	0.706(0.395-1.262)	0.241	0.963(0.389-2.385)	
N stage[3]			0.956		0.395
	N1	0.935(0.559-1.563)	0.797	2.805(0.761-10.346)	0.121
	N2	1.113(0.607-2.041)	0.730	3.795(0.830-17.350)	0.086
	N3	0.905(0.321-2.552)	0.850	3.654(0.602-22.173)	0.159
AJCC stage[4]			0.001*		0.008*
	2	0.131(0.043-0.402)	0.000*	0.115(0.028-0.470)	0.003*
	3	0.129(0.043-0.392)	0.000*	0.035(0.003-0.355)	0.005*
Tumor size			0.753		0.523
	>5 cm	1.083(0.659-1.778)	0.753	1.209(0.675-2.166)	0.523
CCL2 expression			0.014		0.013
	high	1.729(1.115-2.681)	0.014*	1.884(1.141-3.112)	0.013*

1 Clinical stage was divided into I, II, and III; stage II and III were compared to stage I, respectively.

2 T stage was divided into T1 or T2, T3 or T4; T1 or T2 were set as reference.

3 N stage was divided into N0\N1\N2\N3; N1\N2\N3 were compared to N0 respectively.

4 AJCC stage was divided into 1\2\3. Stage 2 and 3 were compared to stage 1, respectively.

Supplementary table S6. KEGG pathway enrichment by GSEA analysis

PATHWAY NAME	SIZE	ES	NES	NOM p-val	FDR q-val	FWER p-val
KEGG_TGF_BETA_SIGNALING_PATHWAY	85	0.610	1.976	0.000	0.086	0.062
KEGG_T_CELL_RECEPTOR_SIGNALING_PATHWAY	108	0.613	1.922	0.000	0.048	0.091
KEGG_CHEMOKINE_SIGNALING_PATHWAY	188	0.622	1.921	0.000	0.042	0.095
KEGG_TOLL_LIKE_RECEPTOR_SIGNALING_PATHWAY	102	0.573	1.887	0.000	0.042	0.124
KEGG_PATHWAYS_IN_CANCER	325	0.459	1.877	0.000	0.044	0.144
KEGG_CYTOKINE_CYTOKINE_RECEPTOR_INTERACTION	264	0.652	1.843	0.000	0.043	0.186
KEGG_FOCAL_ADHESION	198	0.579	1.818	0.000	0.038	0.228
KEGG_HEMATOPOIETIC_CELL_LINEAGE	84	0.725	1.784	0.000	0.045	0.282
KEGG_PRION_DISEASES	35	0.603	1.753	0.000	0.051	0.354
KEGG_COMPLEMENT_AND_COAGULATION_CASCADES	68	0.635	1.709	0.000	0.061	0.468
KEGG_HYPERTROPHIC_CARDIOMYOPATHY_HCM	83	0.612	1.670	0.000	0.064	0.556
KEGG_MAPK_SIGNALING_PATHWAY	265	0.432	1.666	0.002	0.064	0.568
KEGG_ADHERENS_JUNCTION	73	0.471	1.943	0.002	0.073	0.079
KEGG_REGULATION_OF_ACTIN_CYTOSKELETON	210	0.486	1.839	0.002	0.038	0.192
KEGG_CELL_ADHESION_MOLECULES_CAMS	131	0.666	1.731	0.002	0.052	0.411
KEGG_ACUTE_MYELOID_LEUKEMIA	57	0.469	1.925	0.002	0.060	0.090
KEGG_DILATED_CARDIOMYOPATHY	90	0.605	1.610	0.002	0.070	0.705
KEGG_INTESTINAL_IMMUNE_NETWORK_FOR_IGA_PRODUCTION	46	0.747	1.706	0.002	0.060	0.472
KEGG_CHRONIC_MYELOID_LEUKEMIA	73	0.441	2.014	0.002	0.104	0.041
KEGG_B_CELL_RECEPTOR_SIGNALING_PATHWAY	75	0.578	1.841	0.002	0.041	0.189
KEGG_NATURAL_KILLER_CELL_MEDIATED_CYTOTOXICITY	132	0.598	1.747	0.002	0.049	0.369
KEGG_NOD_LIKE_RECEPTOR_SIGNALING_PATHWAY	62	0.647	1.853	0.004	0.044	0.174
KEGG_FC_GAMMA_R_MEDIATED_PHAGOCYTOSIS	95	0.512	1.892	0.004	0.045	0.115
KEGG_JAK_STAT_SIGNALING_PATHWAY	155	0.506	1.654	0.004	0.065	0.604
KEGG_ECM_RECEPTOR_INTERACTION	83	0.642	1.673	0.004	0.066	0.552
KEGG_TYPE_I_DIABETES_MELLITUS	41	0.767	1.706	0.004	0.058	0.472
KEGG_ALLOGRAFT_REJECTION	35	0.777	1.619	0.004	0.070	0.687
KEGG_GRAFT_VERSUS_HOST_DISEASE	37	0.801	1.657	0.004	0.067	0.600
KEGG_VIRAL_MYOCARDITIS	68	0.635	1.767	0.004	0.049	0.324
KEGG_MELANOMA	71	0.512	1.606	0.006	0.070	0.713
KEGG_PANCREATIC_CANCER	70	0.399	1.790	0.006	0.045	0.272
KEGG_LEISHMANIA_INFECTION	70	0.692	1.819	0.006	0.041	0.228
KEGG_AUTOIMMUNE_THYROID_DISEASE	50	0.697	1.674	0.006	0.068	0.550
KEGG_MTOR_SIGNALING_PATHWAY	52	0.417	1.744	0.008	0.048	0.382
KEGG_RENAL_CELL_CARCINOMA	70	0.418	1.830	0.008	0.039	0.207
KEGG_ASTHMA	28	0.764	1.672	0.008	0.065	0.554
KEGG_ENDOCYTOSIS	180	0.363	1.748	0.008	0.051	0.367
KEGG_ANTIGEN_PROCESSING_AND_PRESENTATION	81	0.640	1.684	0.009	0.065	0.523
